# Peroxisome Proliferator-Activated Receptor (PPAR) Agonists in Chronic Liver Diseases: Translating Mechanistic Insights into Clinical Practice and Future Perspectives

**DOI:** 10.3390/cells15141292

**Published:** 2026-07-19

**Authors:** Mario Romeo, Claudio Basile, Andrea Imperatore, Giambattista Mozzi, Fiammetta Di Nardo, Carmine Napolitano, Paolo Vaia, Luigi Di Puorto, Mattia Indipendente, Marcello Dallio, Alessandro Federico

**Affiliations:** Hepatogastroenterology Division, Department of Precision Medicine, University of Campania Luigi Vanvitelli, Piazza Miraglia 2, 80138 Naples, Italy; mario.romeo@unicampania.it (M.R.); claudio.basile@unicampania.it (C.B.); andrea.imperatore@studenti.unicampania.it (A.I.); giambattista.mozzi@studenti.unicampania.it (G.M.); fiammetta.dinardo@unicampania.it (F.D.N.); carmine.napolitano@unicampania.it (C.N.); paolo.vaia@unicampania.it (P.V.); luigi.dipuorto2@studenti.unicampania.it (L.D.P.); mattia.indipendente@studenti.unicampania.it (M.I.); alessandro.federico@unicampania.it (A.F.)

**Keywords:** precision medicine, metabolic dysfunction, cholestatic liver disease

## Abstract

Peroxisome proliferator-activated receptors (PPARα, PPARβ/δ, and PPARγ) are ligand-activated nuclear transcription factors that orchestrate key metabolic and immuno-inflammatory networks governing hepatic homeostasis. By regulating fatty acid oxidation, insulin signaling, bile acid metabolism, and hepatic stellate cell activation, PPARs integrate metabolic and inflammatory cues central to the pathogenesis of chronic liver disorders. Chronic liver diseases (CLDs) constitute a major and growing global health burden. Metabolic dysfunction-associated steatotic liver disease (MASLD), now affecting up to one-third of the adult population worldwide, is closely linked to type 2 diabetes, cardiovascular disease, and major liver-related events. In parallel, chronic immune-mediated cholestatic liver diseases continue to pose important therapeutic challenges. Although ursodeoxycholic acid remains the standard first-line therapy for primary biliary cholangitis (PBC), and several second-line therapeutic options are now available, a substantial proportion of patients exhibit an incomplete biochemical response, while effective disease-modifying therapies for primary sclerosing cholangitis (PSC) remain lacking. Across etiologies, persistent metabolic stress, immune-mediated injury, and maladaptive fibrogenesis represent convergent pathogenic pathways. In MASLD, PPAR agonists have shown promising effects on steatosis, necroinflammatory activity, and fibrosis regression in randomized clinical trials, positioning them among the most advanced pharmacological strategies currently under investigation. In cholestatic liver diseases, selective and dual PPAR agonists have demonstrated significant improvements in cholestasis, pruritus, and markers of disease activity, supporting their role as second-line or adjunctive therapy. This review critically appraises the current preclinical and clinical evidence on the role of PPARs in CLDs, delineates the underlying molecular mechanisms, and discusses future therapeutic perspectives. Although the available evidence is encouraging, most clinical studies have primarily demonstrated improvements in surrogate biochemical and histological endpoints rather than hard clinical outcomes. Ongoing phase III trials and long-term outcome studies will be essential to define the role of PPAR agonists within future therapeutic algorithms for CLDs.

## 1. Introduction

Chronic liver diseases (CLDs) represent a major and increasingly prevalent global health challenge, accounting for a substantial and steadily rising burden of morbidity, mortality, and healthcare utilization worldwide [1]. Beyond their impact on survival, these disorders are characterized by progressive fibrogenesis, which represents the main determinant of long-term prognosis, and are associated with major liver-related complications, including hepatic decompensation, hepatocellular carcinoma (HCC), and the need for liver transplantation [1].

CLDs comprise a heterogeneous group of disorders that can be classified according to their underlying etiology into viral hepatitis, metabolic dysfunction-associated liver disease (MASLD), alcohol-related liver disease (ALD), immune-mediated liver diseases (including autoimmune hepatitis and immune-mediated cholestatic disorders), drug-induced liver injury, and inherited genetic disorders [2,3]. Historically, chronic hepatitis B virus (HBV) and hepatitis C virus (HCV) infections represented the leading causes of advanced chronic liver disease [3]. Over the past decades, however, widespread HBV vaccination programs and the introduction of highly effective antiviral therapies have substantially reduced the burden of viral liver disease in many regions of the world [2,3]. At the same time, profound lifestyle changes—including increasing physical inactivity, calorie-dense diets, obesity, and harmful alcohol consumption—have driven a marked rise in MASLD and ALD, progressively reshaping the global epidemiology of CLDs [4]. In particular, MASLD is currently the most common cause of chronic liver damage worldwide [5], with an estimated global prevalence of 38%, and future projections increasing to 55.4%, driven by the growing prevalence of obesity and type 2 diabetes mellitus (T2DM) [6,7]. In this context, the growing prevalence of metabolic risk factors, harmful alcohol use, and population aging has further amplified their clinical and public health relevance, underscoring the urgent need for effective disease-modifying therapies capable of targeting the core pathogenetic mechanisms [1].

In parallel, immune-mediated cholestasis, a group of chronic diseases encompassing Primary Biliary Cholangitis (PBC), Primary Sclerosing Cholangitis (PSC), and IgG4-Related Sclerosing Cholangitis, constitutes other emerging causes of CLD [8]. Among these, PBC and PSC represent two immune-mediated cholangiopathies with distinct therapeutic landscapes. In PBC, ursodeoxycholic acid (UDCA) remains the standard first-line therapy, while second-line therapeutic options are now available for patients with an inadequate biochemical response. Conversely, effective disease-modifying pharmacological therapies for PSC remain lacking. Despite these advances, both diseases may progress to fibrosis, cirrhosis, portal hypertension, and liver failure, while substantially impairing quality of life because of debilitating symptoms such as fatigue and pruritus [8].

Despite their heterogeneous etiologies, CLDs share common pathophysiological pathways that drive chronic inflammation and fibrogenesis, thereby driving disease progression and ultimately delineating targets for mechanism-based therapeutic interventions. Disease progression is driven by multiple interconnected processes, including metabolic dysfunction, chronic inflammation, and activation of hepatic stellate cells (HSCs), ultimately resulting in extracellular matrix deposition and the development of fibrosis, which is the main prognostic determinant in patients with CLD [9,10]. Persistent inflammation represents the major driver of progression toward fibrosis, and activation of toll-like receptors (TLRs) is considered a key triggering mechanism [11]. In this context, HSCs are activated through the action of multiple mediators released by neighbouring cells, including transforming growth factor beta-1 (TGFB1), reactive oxygen species (ROS), platelet-derived growth factor (PDGF), and angiotensin II [12,13,14,15]. Once activated, HSCs produce enzymes such as lysyl oxidase (LOX), lysyl oxidase-like proteins (LOXLs), and transglutaminases, all of which mediate collagen cross-linking and deposition [12,13,14,15,16].

Furthermore, activated HSCs express tissue inhibitors of metalloproteinases, thereby reducing extracellular matrix degradation [12]. With the persistence of fibrogenic stimuli, as occurs in chronic viral hepatitis, metabolic syndrome-associated inflammation, and autoimmune liver diseases, HSCs remain activated, resulting in excessive extracellular matrix deposition that alters hepatic architecture and elasticity, ultimately increasing the risk of liver failure. Interestingly, emerging evidence suggests the reversibility of the process, sustaining the regression of fibrosis when the underlying triggers are removed [14,15,16,17].

Despite remarkable advances in recent years—including the approval of disease-modifying therapies for selected patients with MASH and the expansion of second-line treatment options for PBC—important unmet therapeutic needs remain [14,15,16]. These advances include the recent accelerated approval of the selective thyroid hormone receptor-β agonist resmetirom for adults with non-cirrhotic MASH and stage F2–F3 fibrosis, highlighting the transition toward mechanism-based pharmacological therapies [14,15,16]. Effective strategies capable of preventing or reversing advanced hepatic fibrosis across different CLDs are still lacking, and no disease-modifying pharmacological therapy has yet been approved for PSC. These limitations continue to drive the search for therapeutic targets capable of simultaneously modulating multiple pathogenic pathways involved in CLD progression [13,14]. In this context, Peroxisome Proliferator-Activated Receptors (PPARs) are emerging as promising therapeutic candidates owing to their pleiotropic role in the regulation of lipid metabolism, inflammation, and hepatic fibrogenesis [16,17,18,19].

Through coordinated regulation of these interconnected pathways, PPAR agonists have the potential to exert disease-modifying effects across a broad spectrum of liver disorders.

The present review aims to provide a comprehensive and clinically oriented overview of the role of PPAR signaling in chronic liver diseases. We first summarize the biological functions and tissue-specific actions of the three PPAR isoforms, then critically examine the preclinical and clinical evidence supporting the use of selective, dual, and pan-PPAR agonists in major liver disease settings, with particular emphasis on metabolic dysfunction-associated steatotic liver disease and chronic immune-mediated cholestatic disorders. Finally, we discuss current limitations, safety considerations, and future perspectives regarding the therapeutic positioning of PPAR agonists within the evolving landscape of precision hepatology.

## 2. Literature Search Strategy and Study Selection

This narrative review was conducted through a comprehensive literature search to identify the most relevant evidence regarding the biological role of peroxisome proliferator-activated receptors (PPARs) and the therapeutic application of PPAR agonists in CLDs. The electronic databases PubMed/MEDLINE, Scopus, and Web of Science were systematically searched from database inception through June 2026.

The search strategy combined Medical Subject Headings (MeSH) terms and free-text keywords related to PPAR biology and chronic liver diseases, including “PPAR”, “peroxisome proliferator-activated receptor”, “PPAR agonists”, “PPARα”, “PPARγ”, “PPARβ/δ”, “fenofibrate”, “bezafibrate”, “pemafibrate”, “pioglitazone”, “elafibranor”, “seladelpar”, “saroglitazar”, and “lanifibranor”, in combination with “chronic liver disease”, “MASLD”, “MASH”, “NAFLD”, “NASH”, “primary biliary cholangitis”, “primary sclerosing cholangitis”, “cholestatic liver disease”, “liver fibrosis”, “cirrhosis”, and “liver inflammation”.

Priority was given to high-quality evidence, including randomized controlled trials, prospective and observational clinical studies, systematic reviews, meta-analyses, international clinical practice guidelines, and landmark mechanistic studies published in peer-reviewed journals. Additional relevant publications were identified through manual screening of the reference lists of key articles.

Studies not directly related to the role of PPARs in chronic liver diseases, duplicate publications, conference abstracts without full-text availability, editorials, and non-English articles were generally excluded unless they provided essential mechanistic or historical insights relevant to the scope of this review. The final selection of studies was based on their scientific quality, methodological rigor, originality, and relevance to the topics discussed. Given the narrative nature of this review, no formal systematic review protocol or PRISMA-guided study selection process was applied. Instead, the literature was critically selected and synthesized to provide a comprehensive, up-to-date, and clinically oriented overview of current knowledge on PPAR biology and PPAR-targeted therapies in CLDs.

## 3. Biology of PPARs in Hepatic and Extrahepatic Physiology

### 3.1. Structure, Classification, and Transcriptional Regulation of PPARs

Peroxisome proliferator-activated receptors (PPARs) constitute a group of lipid-activated transcription factors belonging to the superfamily of nuclear hormone receptors, with key regulatory functions in energy metabolism. To date, three major PPAR isoforms have been identified: PPARα, PPARβ/δ, and PPARγ [15], each characterized by distinct tissue distribution and partially overlapping biological functions [15].

From a molecular biology perspective, their function is mediated through the modulation of specific target gene expression according to the recruitment of co-activators or co-repressors [15]. This results in complex regulation of cellular proliferation, differentiation, and survival [16]. Structurally, PPARs share the canonical modular organization of nuclear receptors, including an N-terminal activation domain (AF-1), a central DNA-binding domain (DBD), a hinge region, and a C-terminal ligand-binding domain (LBD) containing the activation function AF-2 [17]. Following ligand binding, PPARs undergo conformational changes that promote heterodimerization with the retinoid X receptor (RXR), enabling binding to specific DNA sequences termed peroxisome proliferator response elements (PPREs) located within the promoter regions of target genes [17]. Ligand-dependent activation facilitates the dissociation of co-repressor complexes such as Nuclear Receptor Co-repressor (NCoR) and silencing mediator of retinoid and thyroid hormone receptors (SMRT) and the recruitment of transcriptional co-activators, including steroid receptor co-activator-1 (SRC-1) and CREB-binding protein/p300 co-activator complex (CBP/p300), ultimately promoting chromatin remodeling and transcriptional activation of genes involved in fatty acid oxidation, glucose metabolism, inflammatory regulation, and fibrogenesis [17,18]. In addition to this canonical transactivation pathway, PPARs exert important trans-repressive functions through interference with pro-inflammatory transcription factors such as nuclear factor kappa-light-chain-enhancer of activated B cells NF-κB, activator protein-1 (AP-1), and signal transducer and activator of transcription (STAT) pathways [17]. These anti-inflammatory effects occur through multiple mechanisms, including protein–protein interactions, sequestration of co-activators, and stabilization of repressive transcriptional complexes [17].

Finally, PPAR activity is further modulated by post-translational modifications, including phosphorylation, ubiquitination, and SUMOylation, which dynamically regulate receptor stability, cofactor recruitment, DNA binding affinity, and transcriptional activity independently of ligand binding [19].

Collectively, these mechanisms position PPARs as central integrators of metabolic and inflammatory signaling pathways, coordinating adaptive cellular responses under physiological and pathological conditions [20].

### 3.2. PPARs: Overviewing Isoform-Specific Tissue Distribution and Functions

#### 3.2.1. Pleiotropic Roles of PPARα: Hepatic and Extrahepatic Implications in Human Health

PPARα is predominantly expressed in metabolically active tissues characterized by high rates of fatty acid oxidation, including the liver, heart, skeletal muscle, brown adipose tissue, and intestinal mucosa, highlighting the major contribution of these receptors to energy combustion processes [21]. Consistent with this, the principal modulators of PPARα activity include hormones (insulin and glucocorticoids), nutritional status, and endogenous ligands such as mono- and polyunsaturated fatty acids and eicosanoids [22].

Interestingly, unlike many other nuclear receptors, ligand binding to PPARα appears to induce relatively limited conformational rearrangements, primarily stabilizing receptor conformations already predisposed to interaction with regulatory proteins, as demonstrated by biochemical, crystallographic, and co-regulator recruitment studies [23,24]. In line with this, murine models, Qi C et al. demonstrated that PPARα activation does not always rely on a uniform underlying mechanism, although indirect confirmation emerges from responses to synthetic agonists such as fibrates, widely employed in clinical practice as regulators of lipid gene expression [25]. In a seminal study, Chakravarthy et al. demonstrated that liver-specific inactivation of the fatty acid synthase (FAS) gene in mice results in a phenotype identical to that observed in fasting PPARα-deficient mice [26]. These findings suggest that de novo synthesized FAS products, such as palmitate, regulate PPARα activity and that defects in fatty acid β-oxidation and cholesterol metabolism may potentially be corrected through PPARα agonist therapy [26].

In line with this, PPARα has been demonstrated as a cornerstone receptor in regulating downstream expression of enzymes and apolipoproteins involved in triglyceride and cholesterol metabolism and transport, including lipoprotein lipase (LPL), apolipoprotein C-III (ApoC-III), and apolipoprotein A-I (ApoA-I), thereby promoting reductions in small dense LDL particles (sdLDL) and enhancing formation of HDL particles that facilitate reverse cholesterol transport and hepatic cholesterol clearance [27]. Staels B et al. demonstrated in both human and murine hepatocytes, in vitro and in vivo, that PPARα agonists reduce ApoC-III synthesis, an inhibitor of LPL activity, thereby favoring VLDL lipolysis and generation of larger LDL particles more efficiently cleared via the LDL receptor [28].

Considering this, the role of PPARα in atherosclerosis pathogenesis is extensively documented by numerous cellular and clinical studies [28,29,30]. Within this complex context, PPARα emerges as a key modulator with both anti- and pro-atherogenic effects depending on cellular context and inflammatory state. In vitro studies, further supported by clinical evidence, suggest a predominantly protective role, since PPARα activation inhibits expression of inflammatory mediators, such as monocyte chemoattractant protein-1 (MCP-1), Interleukin-6 (IL-6), Vascular Cell Adhesion Molecule protein 1 (VCAM-1), and endothelins, while also reducing IFN-γ expression by T lymphocytes [29,30].

Another particularly relevant aspect concerns cholesterol metabolism within macrophages. PPARα promotes cholesterol efflux through the induction of ABCA1 and ABCG1 transporters, thereby enhancing reverse cholesterol transport (RCT). Furthermore, it regulates proteins involved in intracellular cholesterol trafficking, including NPC1 and NPC2, collectively contributing to the reduction in the intracellular lipid burden [31]. Overall, PPARα receptors exert profoundly beneficial effects on the entire atherogenic process as well as subsequent plaque progression and potential acute events caused by plaque enlargement or rupture. Despite these findings, animal studies have produced conflicting results. In genetically modified murine models such as ApoE−/− or LDL-R−/− mice, PPARα deficiency or activation does not consistently produce overlapping effects [32,33].

Studies conducted by Tordjman K et al. demonstrated that PPARα deficiency protects mice against atherosclerosis, suggesting a potential pro-atherogenic role of the receptor. However, ApoE−/− mice fed a Western diet develop atherosclerotic lesions that regress moderately following fenofibrate treatment, an effect further enhanced in ApoE−/− strains carrying the human ApoA1 transgene. Conversely, marked reductions in the formation and size of pre-existing atherosclerotic lesions have been observed in LDL receptor-deficient mice and ApoE2 knock-in mice treated with GW7647, a highly selective PPARα agonist [34,35]. Although these examples reveal substantial discrepancies between knockout models and agonist treatment studies, an important confounding factor relates to the distinct pharmacokinetic properties of PPARα ligands in murine versus human models, particularly considering interspecies differences in lipid metabolism and transport.

Consequently, caution is warranted when extrapolating data regarding the effects of PPARα on atherosclerotic disease, given the species-specific functional and ligand-binding properties [36,37].

Clinically, numerous studies have evaluated fibrates, as selective PPARα agonists, in cardiovascular prevention.

Trials such as the Helsinki Heart Study, the FIELD study, and the Bezafibrate Infarction Prevention trial demonstrated that these drugs improve lipid profiles and reduce cardiovascular events in patients with metabolic syndrome, type 2 diabetes, and atherogenic dyslipidemia, and thus indirectly in the MASLD and hepatic disease context [37,38,39].

PPARα represents the predominant PPAR isoform at the hepatic level, where it exerts a central role in regulating lipid metabolism and energy homeostasis [38,39].

The high expression of PPARα in the liver, compared with other tissues, reflects the primary role of this organ in coordinating systemic metabolic fluxes, particularly during fasting or energetic stress conditions [40,41]. Gene expression studies and transcriptomic analyses have confirmed that PPARα is abundantly expressed in hepatocytes, whereas other isoforms, such as PPARγ, remain marginal and are more relevant within adipose tissue [42,43]. Functionally, PPARα acts as a transcriptional regulator of a broad network of genes involved in fatty acid β-oxidation, lipid transport, and ketogenesis, consolidating the liver as the main site of lipid oxidation. Activation of PPARα induces coordinated expression of mitochondrial and peroxisomal enzymes, including carnitine palmitoyltransferase 1A (CPT1A), acyl-CoA oxidase 1 (ACOX1), and medium-chain acyl-CoA dehydrogenase (MCAD), thereby promoting the use of fatty acids as an energy source [43,44,45]. This regulatory framework is particularly evident during fasting, a condition in which PPARα is activated by endogenous ligands derived from lipolysis, facilitating hepatic metabolic adaptation. Models with hepatocyte-specific deletion have demonstrated that hepatic loss of PPARα alone is sufficient to induce systemic alterations in lipid metabolism and inflammation, underscoring the liver as the predominant functional site of this receptor [44,45]. Beyond lipid metabolism control, PPARα contributes to the regulation of inflammatory responses through transrepressive mechanisms, interfering with pro-inflammatory transcription factors and modulating cytokine and acute-phase gene expression. This dual metabolic and immunomodulatory role further reinforces the relevance of the liver as the principal platform of PPARα action, where nutritional and inflammatory signals are integrated at the transcriptional level [46,47]. Collectively, hepatic predominance of PPARα extends beyond expression patterns and translates into functional specialization, making the liver the main regulatory hub through which this receptor coordinates lipid homeostasis and adaptive responses to metabolic stress.

Beyond its metabolic functions, PPARα receptors are expressed in several immune and vascular wall cell types, where they exert anti-inflammatory and antioxidant activities [48]. In this context, PPARα receptors also exert repressive effects on pro-inflammatory pathways, particularly NF-κB and AP-1 signaling, while reducing the expression of genes such as IL-6, COX-2, and VCAM-1, as demonstrated in vitro in endothelial cells and macrophages [48].

Supporting these anti-inflammatory properties, it has long been recognized that patients treated with fibrates (including fenofibrate and pemafibrate, both selective PPARα agonists) exhibit significant reductions in circulating C-reactive protein (CRP) levels [22]. Overall, PPARα emerges as a central metabolic and immunomodulatory regulator, particularly within the liver, where it coordinates adaptive responses to energetic and inflammatory stress. While PPARα predominantly regulates hepatic fatty acid oxidation and metabolic adaptation, other PPAR isoforms exert complementary functions in systemic energy homeostasis.

#### 3.2.2. Pleiotropic Roles of PPARβ/δ: Hepatic and Extrahepatic Implications in Human Health

Among the three isoforms, PPARβ/δ is characterized by a more ubiquitous cellular expression pattern and is involved in regulating fatty acid β-oxidation in nearly all tissues in which it is expressed [48,49,50]. Although the identity of true endogenous PPARβ/δ ligands remains uncertain, fatty acids, triglycerides, and prostacyclin represent the most promising candidates. Unlike PPARα (targeted by fibrates) and PPARγ (targeted by glitazones), no clinically approved PPARβ/δ agonists currently exist, although promising compounds such as GW501516 have entered phase II clinical trials for dyslipidemia [49].

Activation of PPARβ/δ has been shown to enhance lipid catabolism in skeletal muscle, heart, and adipose tissue while improving serum lipid profiles and insulin sensitivity in several animal models [50]. Furthermore, PPARβ/δ ligands prevent weight gain and suppress macrophage-derived inflammation [51,52].

Despite promising metabolic effects observed in preclinical and early clinical studies, the clinical development of selective PPARβ/δ agonists remains limited because of unresolved long-term safety concerns, particularly regarding potential pro-tumorigenic effects described in experimental models [53]. About this, effects related to proliferation, angiogenesis, and cell death have also been extensively investigated, particularly because these receptors may be implicated in tumorigenesis, cancer progression, and metastasis across several malignancies, including colorectal cancer [52,53,54].

#### 3.2.3. Pleiotropic Roles of PPARγ: Hepatic and Extrahepatic Implications in Human Health

PPARγ regulates lipid and glucose homeostasis, as supported by recent discoveries of novel PPARγ-regulated genes [54,55]. Two splice isoforms belong to this subgroup: γ1 and γ2. The γ2 isoform is expressed exclusively in adipose tissue, whereas γ1 shows broader expression, although it remains most abundant in adipocytes. The identity of endogenous PPARγ activators remains uncertain, although fatty acids and prostaglandin J2 are among the most extensively studied ligands [56]. Several lines of evidence suggest that PPARγ activation induces insulin sensitization, with highly favorable implications for conditions such as diabetes mellitus and metabolic syndrome [57].

This is supported by observations that ligands structurally related to thiazolidinediones, pharmacological agents acting as selective PPARγ ligands, share in vitro activation properties toward these receptors. More importantly, ligands of the retinoid X receptor (RXR), which can activate the PPARγ-RXR heterodimer, also exert insulin-sensitizing effects in rodents [58]. Supporting these findings, Altshuler D et al., through a case–control study involving three Scandinavian familial cohorts from Sweden and Finland, demonstrated that individuals carrying the Pro12Ala polymorphism in the PPARγ gene, which marginally alters receptor activity, exhibit increased insulin sensitivity [59,60].

Nevertheless, evidence is not entirely consistent, since Kubota et al. demonstrated that mice lacking one allele of the Pparg gene display greater insulin sensitivity than wild-type littermates [61].

In humans, under physiological conditions, hepatic expression of PPARγ is relatively limited; however, significant upregulation occurs in metabolic conditions characterized by insulin resistance and lipid overload, such as MASLD [62]. About this, experimental studies have shown that hepatic activation of PPARγ promotes lipid accumulation through the induction of lipogenic genes, thereby contributing to steatosis [62]. Conversely, clinical evidence suggests that systemic PPARγ activation may indirectly improve hepatic status by reducing the flux of free fatty acids from adipose tissue to the liver and improving overall insulin sensitivity [63]. Overall, PPARγ emerges as a fundamental regulator of the balance between lipid storage and mobilization, exerting pleiotropic effects that reflect the close interconnection between adipose tissue and liver in metabolic pathophysiology.

In parallel, PPARγ exerts major anti-inflammatory effects that reduce insulin resistance and promote lipid storage within adipose tissue, thereby decreasing systemic lipotoxicity. This dual metabolic and immunomodulatory role makes it a fundamental potential therapeutic target in T2DM and related metabolic disorders, including MASLD [64].

Beyond its well-established role in glucose and lipid metabolism, indeed, PPARγ is also a master regulator of adipocyte differentiation and immune cell programming [65]. Activation of PPARγ promotes adipogenesis and enhances the capacity of adipose tissue to safely store lipids, thereby limiting ectopic lipid deposition and systemic lipotoxicity [66]. Moreover, PPARγ contributes to macrophage polarization toward an anti-inflammatory M2 phenotype, attenuates pro-inflammatory cytokine production, and modulates mitochondrial function and oxidative metabolism, collectively supporting cellular metabolic flexibility and tissue homeostasis [67]. These pleiotropic properties further reinforce the central role of PPARγ at the interface between metabolism, immunity, and CLD.

Finally, the hepatic activation status of PPARγ also regulates inflammatory signaling, ultimately playing a particularly relevant role in HSC biology and fibrosis progression. About this, quiescent HSCs physiologically express PPARγ, whereas receptor downregulation accompanies fibrosis activation processes [63]. On the other hand, experimental studies suggest that restoration of PPARγ signaling may partially revert activated HSCs toward a less fibrogenic phenotype, supporting a potential antifibrotic role [68,69].

Collectively, PPARs represent central metabolic and immunological regulators integrating lipid metabolism, inflammation, and fibrogenesis. These pleiotropic functions provide the biological rationale supporting the pivotal role of PPAR signaling pathways in hepatic disorders, particularly in MASLD and cholestatic liver diseases.

**Figure 1:**
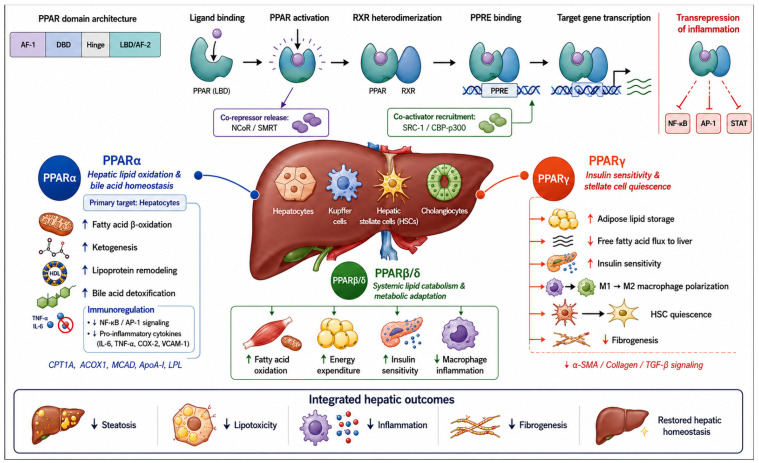
Integrated liver-centered overview of PPAR signaling, illustrating receptor activation, transcriptional regulation, and the isoform-specific biological functions of PPARα, PPARβ/δ, and PPARγ in chronic liver diseases. Following ligand binding, PPARs heterodimerize with RXR, bind to peroxisome proliferator response elements (PPREs), recruit transcriptional co-activators, and regulate the expression of target genes involved in lipid metabolism, bile acid homeostasis, inflammation, insulin sensitivity, and fibrogenesis. In addition to its metabolic functions, PPARα exerts immunoregulatory effects by suppressing NF-κB/AP-1 signaling and reducing the expression of pro-inflammatory mediators. ACOX1, acyl-CoA oxidase 1; AF-1, activation function-1; AP-1, activator protein-1; ApoA-I, apolipoprotein A-I; CBP/p300, CREB-binding protein/p300; CPT1A, carnitine palmitoyltransferase 1A; COX-2, cyclooxygenase-2; DBD, DNA-binding domain; IL-6, interleukin-6; LBD, ligand-binding domain; LPL, lipoprotein lipase; MCAD, medium-chain acyl-CoA dehydrogenase; M1/M2, pro-inflammatory (M1) to anti-inflammatory (M2) macrophage phenotype shift; NCoR, nuclear receptor co-repressor; NF-κB, nuclear factor kappa-light-chain-enhancer of activated B cells; PPAR, peroxisome proliferator-activated receptor; PPARα, peroxisome proliferator-activated receptor alpha; PPARβ/δ, peroxisome proliferator-activated receptor beta/delta; PPARγ, peroxisome proliferator-activated receptor gamma; PPRE, peroxisome proliferator response element; RXR, retinoid X receptor; SMRT, silencing mediator of retinoid and thyroid hormone receptors; SRC-1, steroid receptor co-activator-1; STAT, signal transducer and activator of transcription; TGF-β, transforming growth factor-beta; TNF-α, tumor necrosis factor-alpha; VCAM-1, vascular cell adhesion molecule-1. This figure was created using FigureLabs (https://www.figurelabs.ai; accessed on 24 May 2026) and subsequently reviewed and verified by the authors for scientific accuracy.

Figure 1 summarizes the structural organization of PPARs, their ligand-activated transcriptional and transrepressive mechanisms, and the isoform-specific metabolic, anti-inflammatory, and anti-fibrotic effects they exert across hepatic and extrahepatic tissues (Figure 1).

## 4. Pathogenetic Role of PPARs in Chronic Liver Diseases

Among CLDs, the pathogenetic role of PPAR signaling has been most extensively investigated in MASLD/MASH and chronic immune-mediated cholestatic liver diseases. Accordingly, the following sections focus on these conditions, which currently provide the strongest mechanistic and translational evidence supporting PPAR-targeted therapeutic strategies.

### 4.1. Exploring the Centrality of PPARs in the Pathogenesis of MASLD/MASH

MASLD and its progressive inflammatory form, metabolic dysfunction-associated steatohepatitis (MASH), are currently among the leading causes of CLD worldwide and are closely associated with obesity, insulin resistance, T2DM, and metabolic syndrome [70]. Considering that many of the appropriate therapeutic targets for these diseases remain incompletely characterized, the current challenge is the identification of the molecular mechanisms underlying these conditions in order to develop targeted pharmacological approaches aimed at their modulation [66,67,68].

The pathogenesis of MASLD/MASH remains complex, representing a multifactorial scenario characterized by the interplay between lipid accumulation, metabolic stress, inflammation, oxidative injury, and progressive fibrogenesis [67,68,69,70].

In this heterogeneous context, a central common mechanism is represented by the dysregulation of hepatic lipid metabolism, leading to excessive accumulation of triglycerides and toxic lipid intermediates within hepatocytes [67,68,69].

Under physiological conditions, the PPARs, particularly the PPARα isoform, play a crucial role in preserving hepatic lipid homeostasis by promoting fatty acid β-oxidation, ketogenesis, and triglyceride turnover [71,72]. Supporting this, Patsouris et al. demonstrated in murine models that a high-fat diet (HFD) induces different responses in wild-type mice compared to PPARα knockout mice: in wild-type animals, HFD increases hepatic PPARα mRNA expression and that of downstream target genes such as CYP4A10 and CYP4A14, which are involved in lipid turnover and fatty acid β-oxidation, whereas in PPARα-deficient mice, HFD leads to increased circulating triglycerides and a higher predisposition to dysmetabolic liver disease [73]. Consistently, in the hepatic steatosis context, a reduced PPARα expression or activity, resulting in decreased hepatic oxidative capacity and subsequent lipid accumulation within hepatocytes, has been reported [74,75].

In parallel, pathological conditions such as obesity and metabolic syndrome promote upregulation of PPARγ, particularly in the liver, where its basal expression is relatively low compared to other PPAR isoforms, leading to induction of lipogenic genes and intracellular triglyceride accumulation, thereby exacerbating steatosis and promoting progression toward MASH [72,73,74].

In this picture, insulin resistance emerges as the real driver of liver disease progression in MASLD [76,77]. Insulin resistance enhances adipose tissue lipolysis, increasing the flux of free fatty acids to the liver, where they accumulate and exert lipotoxic effects [74,75,76]. In addition, hepatic insulin resistance disrupts the regulation of gluconeogenesis and lipogenesis, creating a dysfunctional metabolic environment characterized by hypertriglyceridemia and hyperglycaemia [74,75,76]. Within this context, PPAR, particularly PPARγ and PPARα, play a complementary, controversial role.

PPARγ improves systemic insulin sensitivity by modulating adipocyte metabolism and contributing to the stabilization of lipid flux between adipose tissue and liver [78,79]. Experimental evidence has shown that loss of PPARγ in leptin-deficient individuals improves hepatic lipid profiles by reducing lipogenesis and intracellular triglyceride content but markedly worsens glycemic control due to decreased insulin sensitivity in muscle and adipose tissue, thereby aggravating type 2 diabetes mellitus [78,79]. PPARα, in turn, indirectly improves insulin resistance by enhancing fatty acid oxidation and reducing hepatic lipotoxic stress while also regulating genes involved in lipid transport and intracellular lipid handling [80].

Progression from isolated steatosis to MASH is further characterized by activation of inflammatory and oxidative stress pathways. In the MASLD scenario, hepatocellular lipid overload promotes mitochondrial dysfunction, reactive oxygen species (ROS) production, endoplasmic reticulum stress, and ferroptosis, ultimately triggering innate immune activation and inflammatory signaling cascades [76,77]. Relevantly, in this context, PPARα exerts a protective role not only metabolically but also anti-inflammatory, interfering with transcription factors such as NF-κB and reducing the expression of cytokines and chemokines involved in inflammatory cell recruitment [81,82].

Reduced PPARα activity, observed in several MASLD models, is associated with increased hepatic susceptibility to injury and faster disease progression toward MASH [83]. PPARβ/δ and PPARγ also contribute to inflammatory regulation through distinct mechanisms: PPARβ/δ enhances lipid metabolism and reduces systemic inflammation, whereas PPARγ modulates immune cell activity and HSC function, indirectly influencing fibrogenesis [84].

Pharmacological evidence further supports these roles: PPAR agonists such as fibrates and thiazolidinediones have demonstrated, in preclinical and clinical studies, the ability to reduce hepatic lipid content, attenuate inflammation, and, in some cases, improve histological parameters of disease [81,82,83]. Given their integrated effects on metabolism, inflammation, oxidative stress, and fibrogenesis, PPARs represent highly attractive therapeutic targets in MASLD/MASH (as discussed in the subsequent dedicated paragraph).

### 4.2. Exploring the Centrality of PPARs in the Pathogenesis of Chronic Immune-Mediated Cholestatic Liver Diseases

Cholestatic liver diseases, including PBC and PSC, are characterized by impaired bile formation and/or bile flow, leading to intrahepatic accumulation of toxic bile acids, chronic inflammation, cholangiocyte injury, and progressive fibrogenesis [81,82,83]. Although ursodeoxycholic acid (UDCA) remains the standard first-line therapy for PBC, a substantial proportion of patients exhibit incomplete biochemical response, while effective pharmacological options for PSC remain limited [81,82,83].

These unmet therapeutic needs have stimulated growing interest in the role of PPAR signaling in cholestatic liver injury. In this context, nuclear receptors of the peroxisome proliferator-activated receptor (PPAR) family emerge as key regulators of metabolic and inflammatory processes relevant to cholestatic pathogenesis [85]. More specifically, PPARα acts as a transcription factor regulating genes involved in bile acid synthesis, transport, and detoxification. Its activation induces phase II enzymes, including those responsible for bile acid glucuronidation, thereby facilitating their elimination and reducing intracellular toxicity [82,83,84]. In parallel, PPARα regulates canalicular transporter expression on the hepatocyte biliary membrane, contributing to bile acid pool homeostasis and limiting intrahepatic accumulation [86]. Cindoruk et al. demonstrated that in murine models of bile duct ligation treated with a selective PPARα agonist such as fenofibrate, there is a significant improvement in portal inflammation, hepatocellular necrosis, bile duct injury, and overall cholestatic damage [87]. Moreover, PPARs are closely linked to both lipid and bile acid metabolism, reflecting shared biochemical pathways within hepatocytes [88,89]. Genetic deletion studies have shown that loss of PPAR expression disrupts hepatic metabolic homeostasis, including bile acid synthesis and regulation [90].

PPAR signaling closely interacts with other nuclear receptor pathways involved in bile acid homeostasis, particularly the farnesoid X receptor (FXR), which serves as the master regulator of bile acid synthesis, transport, and enterohepatic circulation [91]. Upon activation by bile acids, FXR suppresses hepatic bile acid synthesis through fibroblast growth factor 19 (FGF19)- and small heterodimer partner (SHP)-mediated inhibition of CYP7A1, while simultaneously promoting canalicular bile acid export and detoxification [91]. These complementary biological functions provide a strong mechanistic rationale for combined or sequential therapeutic approaches in cholestatic liver diseases. Clinical studies further indicate that PPARα agonists such as fibrates (notably bezafibrate) improve the clinical course of cholestatic diseases such as PBC, normalizing cholestatic biomarkers including γ-GT and alkaline phosphatase, particularly in patients who are non-responsive or intolerant to UDCA or to FXR agonists, particularly obeticholic acid (OCA) [87,88,89].

PPAR activation, particularly via PPARα and PPARγ, also plays a crucial role in modulating inflammatory responses in cholangiopathies through interference with NF-κB and AP-1 signaling pathways [87,88,89]. Experimental co-culture models have shown that inhibition of PPARγ (e.g., via GW9662) leads to increased secretion of pro-inflammatory cytokines in inflammatory conditions such as inflammatory bowel disease (IBD) and autoimmune cholangiopathies [92].

Additionally, in PBC, downregulation of hepatic PPARα is associated with overexpression of inflammatory microRNAs (miR-155 and miR-21), linking impaired PPAR signaling to biliary inflammation [88,89,90]. Importantly, treatment with UDCA or lithocholic acid partially reverses these effects in vitro [93].

Overall, clinical and translational studies indicate that PPAR agonists, including fibrates and thiazolidinediones, improve biochemical and cholestatic parameters in patients with cholangiopathies, supporting their anti-inflammatory therapeutic potential [89,90,91].

Collectively, these findings suggest that PPAR signaling represents a biologically plausible therapeutic target in cholangiopathies through the integrated modulation of bile acid metabolism, inflammatory signaling, and fibrogenic pathways.

**Figure 2:**
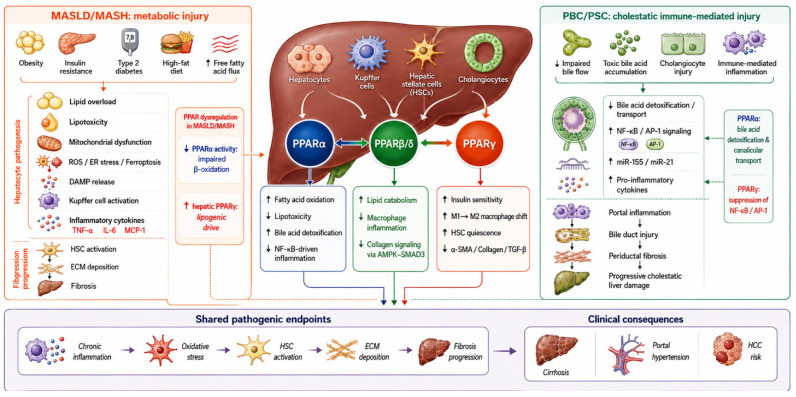
Pathogenetic role of PPAR signaling in MASLD/MASH and chronic immune-mediated cholestatic liver diseases (PBC/PSC). AMPK, AMP-activated protein kinase; AP-1, activator protein-1; DAMP, damage-associated molecular pattern; ECM, extracellular matrix; HCC, hepatocellular carcinoma; IL-6, interleukin-6; MASLD, metabolic dysfunction-associated steatotic liver disease; MASH, metabolic dysfunction-associated steatohepatitis; miR-21, microRNA-21; miR-155, microRNA-155; NF-κB, nuclear factor kappa-light-chain-enhancer of activated B cells; PBC, primary biliary cholangitis; PPAR, peroxisome proliferator-activated receptor; PBC, primary biliary cholangitis; PSC, primary sclerosing cholangitis; ROS, reactive oxygen species; SMAD, small mothers against decapentaplegic; TGFB1, transforming growth factor beta 1; TGF-β, transforming growth factor-beta. This figure was created using FigureLabs (https://www.figurelabs.ai; accessed on 24 May 2026) and subsequently reviewed and verified by the authors for scientific accuracy.

Figure 2 summarizes the most relevant PPAR-related pathogenetic mechanisms in MASLD and chronic immune-mediated cholestatic liver diseases (Figure 2).

### 4.3. Anti-Fibrotic and Immunomodulatory Properties of PPARs in Chronic Liver Diseases

Fibrogenesis represents the final common pathway of chronic liver injury, regardless of etiology, and is primarily mediated by activation of HSCs, which transdifferentiate into myofibroblast-like cells producing extracellular matrix proteins in response to chronic inflammatory and metabolic stimuli [94].

Among PPAR isoforms, PPARγ plays a particularly important role in maintaining HSC quiescence. Physiologically, quiescent HSCs express PPARγ, whereas receptor downregulation accompanies fibrogenic activation and increased extracellular matrix production. Experimental studies have demonstrated that pharmacological restoration of PPARγ signaling inhibits HSC activation, reduces collagen synthesis, and partially reverts activated stellate cells toward a less fibrogenic phenotype [89,90,91]. Downregulation of PPARγ has been observed in fibrotic human liver samples and experimental models, and is associated with increased expression of procollagen, α-SMA, and metalloproteinases, which drive fibrogenesis [90,91,92]. Conversely, pharmacological activation of PPARγ inhibits HSC activation and migration and may promote reversion to a quiescent phenotype, suggesting partial reversibility of fibrosis [68,95,96].

PPARα also indirectly modulates HSC activation by reducing lipotoxicity and oxidative stress within the hepatic microenvironment [92,93,94]. Fibrogenesis is driven by multiple pro-fibrotic pathways, including Transforming Growth Factor-beta/Mothers against decapentaplegic homolog (TGF-β/Smad), NF-κB, and Wnt/β-catenin signaling [92,93,94,95,96].

Clinical evidence from trials such as those evaluating pioglitazone in MASH has demonstrated metabolic and histological improvements, although with limited impact on fibrosis regression [97]. Nevertheless, it appears clear how important the use of PPARγ agonist drugs may be in these groups of patients, considering that the prevention of the above-mentioned pathophysiological processes allows the avoidance of activation of fibrogenic mechanisms, which would otherwise lead to even more severe consequences for hepatic function and survival [93,94,95,96]. Subsequently, the PIVENS study, although only to a limited extent, also confirmed a markedly more positive impact of pioglitazone compared with placebo on the histological characteristics of MASH patients [63]. These changes did not emerge as evidence of histological remission of the disease, but rather as stabilization of pathological processes already underway, through the reduction in systemic circulation of biomarkers of hepatic fibrosis such as the N-terminal propeptide of type III procollagen (PIIINP), measured at the beginning and at the end of the clinical trial [63]. On the other hand, PPARα also exerts an antifibrotic effect, mainly through the reduction in lipotoxicity and oxidative stress caused by the accumulation of lipid molecules directly within hepatocytes and indirectly within HSCs [93,94,95]. Furthermore, PPARα suppresses NF-κB activity, reducing the expression of pro-inflammatory cytokines that promote the establishment first of an inflammatory phenotype and subsequently of a fibrotic one [93,94,95].

PPARβ/δ exhibits a more complex role in fibrogenesis, potentially promoting cell survival while also reducing collagen expression through regulation of AMP-activated protein kinase (AMPK) signaling and downstream inhibition of SMAD3 phosphorylation and p300 activity [69,98,99]. Finally, pan-PPAR agonists such as elafibranor have shown promising results in MASH, improving both inflammation and fibrosis-related parameters [100].

Beyond their direct anti-fibrotic effects, PPARs act as key integrators of lipid metabolism and immune signaling in the liver [101,102]. By converting lipid-derived metabolic signals into transcriptional programs, they modulate immune cell activity and reduce hepatocellular lipotoxicity. This limits the release of damage-associated molecular patterns (DAMPs) and attenuates Kupffer cell activation, thereby reducing inflammatory cascades that drive fibrosis progression [98,99,100,101,102].

Overall, PPARs represent promising therapeutic targets in CLDs characterized by immunometabolic dysfunction and persistent fibrogenesis.

## 5. Pharmacological Modulation of PPAR Agonists in Chronic Liver Diseases: From Receptor Selectivity to Clinical Applications

### 5.1. Pharmacological Classification of PPAR Agonists

Pharmacological modulation of PPAR signaling has emerged as one of the most promising therapeutic strategies in CLDs owing to the central role of PPARs in coordinating lipid metabolism, glucose homeostasis, bile acid regulation, inflammation, and fibrogenesis [103]. Rather than targeting a single pathogenic pathway, PPAR agonists exert pleiotropic effects that simultaneously modulate multiple interconnected mechanisms involved in disease progression, thereby providing a strong biological rationale for their therapeutic application across both metabolic and cholestatic liver diseases [99,100,101,102].

According to receptor selectivity, currently available compounds can be classified into three major pharmacological categories: selective agonists, dual agonists, and pan-PPAR agonists. Each class displays distinct biological properties resulting from the specific combination of activated receptor isoforms and therefore exhibits different metabolic, anti-inflammatory, antifibrotic, and cholestatic effects [99,100,101,102].

Selective agonists activate a single PPAR isoform. PPARα agonists (mainly fibrates) predominantly enhance hepatic fatty acid β-oxidation, improve lipoprotein metabolism, and modulate bile acid homeostasis, making them particularly attractive in cholestatic disorders [100,101,102].

Selective PPARγ agonists, represented by thiazolidinediones, primarily improve insulin sensitivity, reduce adipose tissue lipolysis, and modulate HSC activation, thus exerting beneficial effects in metabolic liver disease despite safety limitations related to fluid retention and weight gain [102,103,104,105,106].

Selective PPARβ/δ agonists, particularly seladelpar, have more recently emerged as promising therapeutic agents owing to their capacity to improve lipid utilization while simultaneously attenuating inflammatory signaling [104,105].

Dual PPAR agonists simultaneously activate two receptor isoforms, thereby integrating complementary metabolic pathways [106]. Elafibranor combines PPARα and PPARβ/δ activation, whereas saroglitazar activates PPARα and PPARγ, allowing broader regulation of lipid metabolism, insulin resistance, inflammation, and cholestatic injury than selective compounds [107].

Pan-PPAR agonists activate all three receptor isoforms simultaneously [103]. By targeting the entire PPAR signaling network, these compounds theoretically represent the most comprehensive pharmacological approach currently available, aiming to simultaneously reduce steatosis, improve insulin sensitivity, attenuate inflammation, limit fibrogenesis, and restore metabolic homeostasis [103]. Lanifibranor currently represents the most clinically advanced molecule within this class [108].

The following sections discuss each pharmacological class separately, first describing the underlying mechanisms of action, followed by available clinical evidence across CLDs, and finally addressing safety considerations and current therapeutic positioning.

Because the current clinical development of PPAR agonists is largely concentrated in MASLD/MASH and chronic cholestatic liver diseases, these conditions inevitably receive greater emphasis in the following sections, reflecting the distribution of the available clinical evidence rather than a deliberate restriction of the review scope.

**Figure 3:**
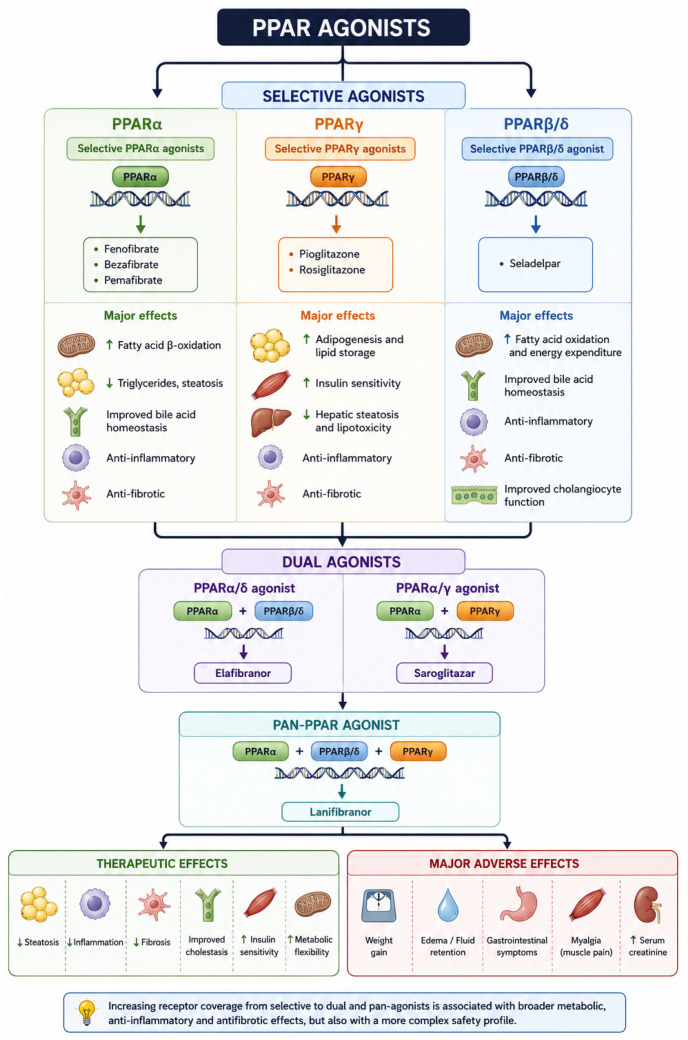
Pharmacological classification, mechanisms of action, and safety profile of PPAR agonists in chronic liver diseases. Selective agonists target a single PPAR isoform (PPARα, PPARγ, or PPARβ/δ), whereas dual agonists simultaneously activate two receptor isoforms (PPARα/δ or PPARα/γ), and pan-PPAR agonists activate all three PPAR isoforms (PPARα/βδ/γ). Increasing receptor coverage is associated with broader metabolic, anti-inflammatory, antifibrotic, and cholestatic effects, while also influencing the spectrum of class-specific adverse events. Representative compounds, principal mechanisms of action, major therapeutic effects, and the most frequently reported adverse events are summarized for each pharmacological class. PPAR, peroxisome proliferator-activated receptor; PPARα, peroxisome proliferator-activated receptor alpha; PPARβ/δ, peroxisome proliferator-activated receptor beta/delta; PPARγ, peroxisome proliferator-activated receptor gamma; FA, fatty acid; β-oxidation, mitochondrial and peroxisomal fatty acid β-oxidation; GI, gastrointestinal. This figure was created using FigureLabs (https://www.figurelabs.ai; accessed on 24 May 2026) and subsequently reviewed and verified by the authors for scientific accuracy.

For clarity, Figure 3 provides an overview of the currently available PPAR agonists, illustrating their receptor selectivity, principal mechanisms of action, therapeutic effects, and the most commonly reported adverse events (Figure 3).

### 5.2. Selective PPAR Alpha Agonists: Exploring the Pleiotropic Role of Fibrates

Selective PPARα agonists represent the oldest and most extensively investigated class of PPAR-targeting compounds [109]. Initially developed for the treatment of atherogenic dyslipidemia, these agents have progressively attracted interest in hepatology owing to the central role of PPARα in regulating hepatic lipid metabolism, bile acid homeostasis, inflammation, and fibrogenesis [109].

By activating genes involved in mitochondrial and peroxisomal fatty acid β-oxidation, PPARα agonists reduce intrahepatic lipid accumulation, improve lipoprotein metabolism, and attenuate lipotoxicity, thereby interrupting key mechanisms underlying chronic liver injury [109].

Beyond these metabolic effects, PPARα activation suppresses NF-κB-mediated inflammatory signaling, reduces pro-inflammatory cytokine production, and modulates bile acid synthesis and transport through transcriptional regulation of enzymes and canalicular transporters involved in bile acid detoxification [100,101,102]. These pleiotropic properties provide the biological rationale for investigating selective PPARα agonists across both metabolic and cholestatic liver diseases [110].

Currently, fenofibrate (a selective PPAR-α agonist) and bezafibrate (with predominant PPARα activity and partial activity toward the other PPAR isoforms) represent the main molecules modulating the PPARα in clinical practice [111].

Fenofibrate is one of the most widely used selective PPARα agonists and has long been employed for the treatment of hypertriglyceridemia and mixed dyslipidemia [100,101,102]. In hepatology, its therapeutic interest derives from its capacity to simultaneously improve hepatic fatty acid oxidation, reduce inflammatory signaling, and enhance bile acid detoxification [100,101,102]. Clinical studies have consistently demonstrated improvements in serum aminotransferases, triglycerides, and markers of hepatic steatosis, whereas evidence regarding fibrosis regression and clinically meaningful liver-related outcomes remains limited [100,101,102]. Consequently, although selective PPARα agonists contribute to metabolic optimization, they are currently considered adjunctive rather than disease-modifying therapies in MASLD/MASH.

Although fenofibrate has demonstrated favorable biochemical effects in dysmetabolic chronic hepatopathies, its greatest clinical experience has been accumulated in cholestatic disorders, particularly PBC, where it has consistently improved cholestatic biochemical markers in patients with incomplete response to UDCA [111]. Several studies have confirmed the beneficial effects of fenofibrate combined with UDCA on biochemical disease markers in PBC [112,113]. However, a large retrospective single-center study published in 2015 by Cheung et al. demonstrated that improvement in ALP levels was not significant, while bilirubin levels increased in cirrhotic patients receiving fenofibrate, suggesting that this agent should be used with great caution in advanced liver disease [111,114,115]. In addition, evidence demonstrated the nephrotoxic potential of fenofibrate through increases in serum creatinine levels [116], potentially due to the PPAR-α-mediated inhibition of prostaglandin-induced renal vasodilation [116].

Among currently available fibrates, bezafibrate has generated the strongest clinical evidence in cholestatic liver diseases. Emerging evidence has demonstrated significant improvements in ALP, bilirubin, and patient-reported symptoms, particularly pruritus, when combined with UDCA in patients with PBC who exhibit an incomplete biochemical response to first-line therapy [117]. Bezafibrate was initially shown in a retrospective study conducted approximately 20 years ago to reduce, and in some cases normalize, hepatobiliary enzymes and total bilirubin levels [110]. More recently, a 24-month randomized, double-blind, placebo-controlled phase III trial enrolled 100 patients with inadequate response to UDCA [117]. Among patients treated with bezafibrate, normalization of ALP was achieved in 67% of cases, compared with 2% in the placebo group [117].

Several studies further demonstrated that combination therapy with UDCA and bezafibrate is associated with improved long-term survival [118,119]. However, dose reduction or discontinuation of bezafibrate was required in some patients because of adverse events, particularly renal dysfunction and myalgia [120,121]. Given the controversial safety profile, fibrates have not yet received formal approval for the treatment of PBC, and their use remains off-label in most countries [122,123].

More recently, the development of selective PPARα modulators (SPPARαMs), such as pemafibrate, has further refined this pharmacological approach by increasing receptor selectivity while minimizing off-target effects, potentially improving the balance between efficacy and tolerability compared with conventional fibrates [100,101,102].

Pemafibrate represents the first selective PPARα modulator (SPPARαM), specifically designed to achieve greater receptor selectivity than conventional fibrates while reducing adverse events associated with off-target receptor activation. Beyond its lipid-lowering properties, pemafibrate has demonstrated favorable effects on hepatic steatosis, insulin resistance, inflammatory activity, and liver enzyme profiles in patients with metabolic dysfunction, suggesting a potential therapeutic role in MASLD [100,101,102]. Although current evidence remains limited compared with fenofibrate and bezafibrate, pemafibrate illustrates the evolution of PPARα pharmacology toward more selective compounds with improved safety profiles.

### 5.3. Selective PPARγ Agonists: Exploring the Pleiotropic Role of Thiazolidinediones

Unlike PPARα agonists, which predominantly enhance hepatic fatty acid oxidation, activation of PPARγ promotes adipocyte differentiation, facilitates lipid storage within adipose tissue, and reduces the flux of free fatty acids to the liver. Consequently, hepatic lipotoxicity is attenuated, leading to improvements in steatosis and metabolic homeostasis [102,103,104,105,106]. Beyond these systemic metabolic effects, PPARγ activation also exerts anti-inflammatory and antifibrotic actions through modulation of HSC activation, promotion of macrophage polarization toward an anti-inflammatory phenotype, and suppression of pro-inflammatory transcription factors, including NF-κB [102,103,104,105,106]. Collectively, these pleiotropic mechanisms provide the biological rationale for the use of selective PPARγ agonists in MASLD/MASH, particularly in patients with obesity, insulin resistance, or diabetes. Although hepatic PPARγ expression is relatively limited under physiological conditions, receptor expression markedly increases in steatotic liver disease, further supporting the concept that pharmacological modulation of PPARγ may modify key metabolic and inflammatory pathways involved in disease progression [102,103,104,105,106].

Currently, thiazolidinediones, including pioglitazone and rosiglitazone, represent the main investigated selective PPAR*γ* agonists [113,114,115]. Given the multifactorial pathogenesis of MASLD/MASH, involving steatosis, lipotoxicity, inflammation, and progressive fibrosis, PPAR agonists offer a mechanistically attractive strategy capable of targeting multiple pathogenic pathways simultaneously. However, the broad systemic distribution of PPARγ also explains the occurrence of several class-specific adverse effects that have limited the widespread clinical adoption of these compounds [102,103,104,105,106].

Rosiglitazone has initially demonstrated metabolic efficacy through selective PPARγ activation [113,114,115]; however, its role in hepatology has progressively declined because of concerns regarding cardiovascular safety and fluid retention. Safety concerns have emerged clearly in long-term cardiovascular studies. The RECORD trial (NCT00379769), which followed 4400 patients treated with rosiglitazone for approximately 5.5 years, demonstrated no increase in cardiovascular mortality compared with controls but showed a doubling in heart failure risk, confirming fluid retention as the major clinical limitation of PPARγ agonism [64].

Consequently, although rosiglitazone contributed substantially to understanding the biological effects of PPARγ agonism, current clinical interest has largely shifted toward pioglitazone and the development of newer selective PPARγ modulators (SPPARγs) designed to preserve efficacy while minimizing adverse events.

Pioglitazone is currently the best-characterized selective PPARγ agonist in hepatology and remains one of the few pharmacological agents consistently demonstrating histological benefit in patients with MASLD/MASH [113,114,115]. Most available evidence derives from improvements in histological activity and metabolic parameters, whereas effects on long-term liver-related clinical outcomes have not yet been established [113,114,115]. The pivotal PIVENS trial first revealed the therapeutic potential of pioglitazone in non-diabetic patients with MASH, demonstrating significant histological improvement compared with placebo [63]. In a systematic review of 25 randomized controlled trials involving 2597 participants (mean BMI 32 ± 3 kg/m^2^; 62% with diabetes), pioglitazone was superior to placebo in improving histological features of MASH without worsening fibrosis, with signals suggesting fibrosis benefit [124]. These findings highlight the central role of PPARγ in integrating metabolism, inflammation, and energy regulation in MASLD/MASH.

In contrast to MASLD/MASH, evidence supporting selective PPARγ agonists in cholestatic liver diseases remains limited. Although experimental studies suggest anti-inflammatory and antifibrotic effects through modulation of HSCs and immune signaling, no convincing clinical evidence currently supports the routine use of selective PPARγ agonists in either PBC or PSC. Therefore, their clinical relevance in cholestatic disorders remains largely mechanistic rather than therapeutic.

### 5.4. Selective PPARβ/δ Agonists: Toward the Modern Precision Targeting

Selective PPARβ/δ agonists represent the most recent class of PPAR-targeting agents introduced into clinical hepatology [104,105]. Compared with PPARα and PPARγ, the biological functions of PPARβ/δ have historically been less extensively investigated; however, growing evidence has highlighted its central role in regulating fatty acid utilization, mitochondrial energy metabolism, inflammatory signaling, and tissue repair [104,105].

Collectively, these pleiotropic effects provide the biological rationale for investigating selective PPARβ/δ agonists in CLDs, particularly those characterized by chronic cholestatic inflammation. Unlike PPARγ agonists, selective PPARβ/δ agonists exert only modest effects on systemic insulin sensitivity and adipose tissue metabolism, while their predominant therapeutic action appears to involve modulation of inflammatory and cholestatic pathways. This pharmacological profile has directed their clinical development primarily toward PBC rather than MASLD/MASH. To date, clinical efficacy has primarily been demonstrated through improvements in biochemical response and patient-reported symptoms, while evidence on long-term clinical outcomes remains limited.

In PBC, Seladelpar represents the most investigated PPAR *β*/*δ* agonist [125,126]. Relevantly, the activation of PPAR-δ by seladelpar induces fibroblast growth factor 21 (FGF21) release from hepatocytes, reducing bile acid accumulation through inhibition of cholesterol 7α-hydroxylase, the rate-limiting enzyme in bile acid synthesis [125,127,128]. These initial findings paved the way to conduct further research in humans.

The first phase II placebo-controlled study evaluating seladelpar enrolled patients with ALP ≥1.67× ULN despite UDCA therapy and randomized them to placebo, seladelpar 50 mg/day, or seladelpar 200 mg/day [127]. Seladelpar produced ALP reductions of up to 60% after 12 weeks, but a threefold increase in aminotransferase levels led to early study termination [127]. Subsequently, Bowlus et al. conducted another phase II study using lower doses (2, 5, or 10 mg/day), with escalation to 10 mg/day in non-responders [129].

After 52 weeks, no patients remained on the 2 mg dose, biochemical response with ALP normalization (<1.67× ULN) and normal bilirubin was achieved in 53% of patients in the 5 → 10 mg group and 69% in the 10 mg group [129]. Only four patients discontinued therapy because of drug-related adverse events, and overall tolerability was favorable [129].

The phase III ENHANCE study further demonstrated that seladelpar 10 mg/day produced significant anticholestatic effects and reduced pruritus in symptomatic patients [130]. More recently, the phase III RESPONSE trial randomized patients in a 2:1 ratio to receive seladelpar 10 mg/day or placebo [125]. The primary endpoint was biochemical response at month 12, defined as ALP <1.67× ULN or reduction ≥15% from baseline, together with normalization of bilirubin and improvement in pruritus [125]. A greater proportion of seladelpar-treated patients achieved biochemical response compared with placebo (61.7% vs. 20%), with significant pruritus improvement (25% vs. 0%).

Adverse event rates were similar between groups (86.0% vs. 84.6%), as were serious adverse events (7% vs. 6.2%) [125]. Long-term efficacy was confirmed by the ASSURE study, which included patients previously enrolled in RESPONSE [131]. Seladelpar maintained biochemical response in 70–73% of patients up to 24 months, with progressive ALP normalization rates reaching 42%. Treatment-naïve patients showed particularly high response rates at 12 months (94%). Significant and sustained improvement in pruritus was also observed, together with stabilization of or reduction in ALT, AST, and GGT [131].

Consistently, the regulatory approval of seladelpar (Livdelzi) was based primarily on improvements in biochemical response, particularly ALP reduction together with bilirubin normalization [129,130]. Although these surrogate endpoints are strongly associated with prognosis in PBC, evidence demonstrating improvements in hepatic decompensation, liver transplantation, or survival is not yet available.

### 5.5. Dual PPAR Agonists: Overcoming the Limitation of Selective Receptor Activation

Dual PPAR agonists were developed to overcome the intrinsic limitations of selective receptor activation by simultaneously modulating complementary metabolic and inflammatory pathways [106]. Whereas selective agonists primarily influence a single biological axis, dual agonists combine the beneficial effects of two PPAR isoforms, thereby targeting multiple interconnected mechanisms involved in CLD progression [106]. This pharmacological strategy is particularly attractive in disorders such as MASLD/MASH and PBC, in which lipid dysregulation, insulin resistance, chronic inflammation, bile acid imbalance, and fibrogenesis coexist and contribute simultaneously to disease progression [106].

In recent years, pharmacological development in MASLD/MASH has progressively shifted toward dual PPAR agonists, which simultaneously activate two receptor isoforms to integrate complementary metabolic and anti-inflammatory effects [70,114,115,116]. Among these, saroglitazar, a potent PPARα agonist and moderate PPARγ agonist, has shown particularly promising results [132]. It was initially introduced in India in 2013 for diabetic dyslipidemia, and its use was later expanded to diabetes management owing to its combined metabolic effects [133]. Saroglitazar has also been approved in India for non-cirrhotic MASH, although not in other countries [134]. In a randomized placebo-controlled phase II study in patients with MASLD/MASH in the United States, saroglitazar 4 mg/day administered for 16 weeks significantly improved serum ALT levels, hepatic fat content, and insulin resistance [70,115,116,117]. These findings suggest a potential therapeutic role, particularly in patients with marked metabolic dysfunction and diabetes [133].

Another dual agonist, elafibranor (PPARα/δ), was evaluated in the phase IIb GOLDEN-505 trial [135]. In this study, non-cirrhotic patients with MASH were randomized to receive elafibranor 80 mg/day, 120 mg/day, or placebo for 52 weeks.

Treatment with elafibranor 120 mg/day showed a favorable trend toward MASH resolution without worsening fibrosis, as confirmed by liver biopsy, while also improving lipid profile, insulin sensitivity, liver enzymes, and inflammatory markers [135]. Importantly, elafibranor demonstrated good tolerability over one year of exposure. However, despite encouraging early results, the subsequent phase III RESOLVE-IT trial failed to meet its primary endpoint, leading to discontinuation of its clinical development for MASH. This highlighted both the biological complexity of MASH and the importance of patient selection [136,137].

Relevantly, since Elafibranor and Saroglitazar also reduce bile acid toxicity and inflammation through downstream modulation of PPAR-α and PPAR-δ signaling pathways, these molecules have been massively investigated in cholestatic scenarios [138].

In PBC, initially, a randomized placebo-controlled phase II study by Schattenberg et al. evaluated elafibranor in patients with inadequate response to UDCA, defined as ALP ≥1.67× ULN (ULN = 104 U/L for women and 129 U/L for men) [139]. Forty-five adults were randomized to receive elafibranor 80 mg, elafibranor 120 mg, or placebo once daily for 12 weeks. Compared with placebo, almost all patients receiving elafibranor achieved a significant reduction in ALP levels (≥20%) [139]. These initial findings provided the rationale for the subsequent phase III ELATIVE trial, a double-blind, placebo-controlled study in which patients with inadequate response or intolerance to UDCA were randomized in a 2:1 ratio to receive elafibranor 80 mg daily or placebo. Biochemical response was observed in 51% of elafibranor-treated patients compared with 4% in the placebo group. The most commonly reported adverse events included abdominal pain, diarrhea, nausea, and vomiting [138]. Elafibranor (Iqirvo) received accelerated approval from the U.S. Food and Drug Administration on 10 June 2024 for the treatment of PBC in combination with UDCA in adults with an inadequate response to UDCA or as monotherapy in patients showing intolerance to UDCA. Subsequently, on 19 September 2024, the European Commission granted conditional marketing authorization following a positive opinion from the European Medicines Agency [140].

Both regulatory approvals were primarily supported by improvements in surrogate biochemical endpoints, particularly ALP reduction and bilirubin normalization, whereas a beneficial effect on hepatic decompensation, liver transplantation, liver-related mortality, or overall survival has not yet been demonstrated. In this sense, since existing evidence is mainly based on improvements in biochemical and histological surrogate endpoints, confirmation of long-term clinical benefit requires ongoing outcome studies.

The current therapeutic positioning of PPAR agonists should also be interpreted in the context of FXR agonism. OCA, a semisynthetic FXR agonist, represented the first approved second-line pharmacological therapy for patients with PBC showing an inadequate response or intolerance to UDCA [141]. Clinical trials demonstrated significant improvements in biochemical cholestatic markers, particularly ALP, supporting its regulatory approval [142,143]. However, its clinical use has been limited by dose-dependent pruritus, safety concerns in patients with advanced cirrhosis, and evolving regulatory recommendations [144,145]. These limitations have contributed to increasing interest in newer therapeutic approaches, including (selective) PPAR agonists, which appear to offer a more favorable tolerability profile while also improving cholestatic symptoms such as pruritus in PBC.

In PSC, compared with PBC, the clinical development of PPAR agonists remains at a much earlier stage, reflecting both the biological complexity of the disease and the persistent lack of effective disease-modifying therapies [146]. Nevertheless, PPAR signaling represents a promising therapeutic target in PSC because of its potential to modulate bile acid homeostasis, cholangiocyte inflammation, immune-mediated injury, and fibrogenesis [146].

In particular, activation of PPARα may enhance bile acid detoxification and transport, whereas PPARδ signaling may exert anti-inflammatory and antifibrotic effects within the cholangiopathic microenvironment [146].

Based on this, elafibranor has recently extended its clinical evaluation to PSC in the phase II ELMWOOD trial [147]. This randomized, placebo-controlled study enrolled 68 patients with PSC and ALP levels ≥1.5× the upper limit of normal, who were assigned to receive elafibranor 80 mg, 120 mg, or placebo once daily for 12 weeks [147]. Treatment with elafibranor was associated with dose-dependent improvements in cholestatic biochemical markers, including reductions in ALP, suggesting a potential anticholestatic effect, while the overall safety profile remained acceptable and broadly consistent with previous studies in cholestatic liver diseases [147]. However, the relatively short treatment duration and limited sample size preclude definitive conclusions regarding long-term clinical efficacy. Therefore, whether these biochemical improvements translate into delayed fibrosis progression, reduced liver-related complications, or improved transplant-free survival remains unknown. At present, no PPAR agonist has received regulatory approval for the treatment of PSC, underscoring the urgent need for larger and longer-term studies to clarify the therapeutic role of PPAR modulation in this challenging disease.

### 5.6. Pan-PPAR Agonists: Toward a Holistic Strategy

Pan-PPAR agonists represent the most comprehensive pharmacological strategy, as they simultaneously activate PPARα, PPARγ, and PPARβ/δ to target lipid metabolism, insulin resistance, inflammation, and fibrosis [103,148]. At the hepatic level, this combined activation enables coordinated reduction in steatosis, stimulation of fatty acid oxidation, and improvement of insulin sensitivity [149,150]. Among these agents, lanifibranor is currently the most extensively studied and clinically advanced compound in MASLD/MASH. Lanifibranor demonstrated beneficial metabolic and antifibrotic effects in preclinical models [103]. Experimental evidence further suggests that pan-PPAR agonists may directly influence fibrosis progression.

Zoe Boyer-Diaz et al. demonstrated that lanifibranor reduces HSC activation and modulates pro-fibrotic cytokine production, with associated improvements in portal hypertension, ascites, inflammation, and fibrosis regression in experimental cirrhosis models [151]. These observations were translated into clinical evidence in the phase IIb NATIVE trial, a randomized double-blind placebo-controlled study evaluating 24-week treatment with lanifibranor (800 mg or 1200 mg daily) in non-cirrhotic adults with MASH [100]. Treatment with lanifibranor 1200 mg resulted in a ≥2-point reduction in the Steatosis Activity Fibrosis (SAF) score without worsening fibrosis [100]. Furthermore, lanifibranor significantly improved necroinflammatory activity and reduced fibrosis compared with placebo, while also improving insulin resistance, lipid profile, and systemic inflammatory markers [152]. These findings strongly support the rationale for pan-PPAR agonism as a therapeutic strategy in advanced MASH [108,152,153]. These encouraging results have led to the ongoing phase III NATiV3 trial, which is currently evaluating the long-term efficacy and safety of lanifibranor in patients with non-cirrhotic MASH and significant fibrosis. Unlike earlier studies, NATiV3 has been specifically designed to provide confirmatory evidence regarding histological efficacy while generating longer-term safety data required for potential regulatory approval. The results of this trial are expected to define the future positioning of pan-PPAR agonism within the therapeutic algorithm of MASLD/MASH [151].

In contrast to MASLD/MASH, at present, clinical evidence supporting pan-PPAR agonists in PBC and PSC remains limited [149,150]. Nevertheless, the simultaneous modulation of bile acid metabolism, inflammation, and fibrosis provides a compelling biological rationale for future evaluation in cholestatic liver diseases [149,150].

Additional clinical studies will be required to determine whether the broad mechanistic profile of lanifibranor translates into clinically meaningful benefits beyond metabolic liver disease.

In this evolving scenario, other emerging pan-PPAR agonists have also demonstrated promising metabolic efficacy. Muraglitazar, for example, showed significant glycemic and lipid improvements in phase II/III trials in diabetes. In a 24-week randomized double-blind trial versus pioglitazone, muraglitazar achieved greater Glycated Haemoglobin (HbA1c) reduction and improved triglyceride and HDL-C levels, although at the cost of weight gain and oedema [154].

Despite these promising results, the principal challenge of multiple-PPAR agonism remains achieving an acceptable safety profile. Simultaneous activation of multiple receptor isoforms requires a delicate pharmacological balance, and several compounds have been abandoned because of undesirable systemic or hepatic adverse effects. Nevertheless, next-generation molecules are demonstrating a more favorable efficacy–tolerability balance.

Moreover, although the available data support meaningful histological improvements, definitive evidence regarding the prevention of hepatic decompensation, liver transplantation, or mortality is still awaited.

Table 1 and Table 2 summarize the most relevant and recent clinical trials investigating PPAR agonists, respectively, in MASLD/MASH (Table 1) and in PBC (Table 2).

## 6. Lights and Shadows of PPAR Pharmacological Modulation in the Management of Chronic Liver Disease: Where Are We?

### 6.1. Overviewing Safety Profiles of PPAR Agonists in MASLD/MASH

The clinical integration of PPAR agonists into the management of CLD—particularly MASLD/MASH—requires a nuanced understanding of isoform-specific safety signals. Although these nuclear receptors offer substantial therapeutic potential through the modulation of hepatic lipid metabolism and inflammatory pathways, their systemic pleiotropic effects necessitate rigorous evaluation of metabolic, cardiovascular, and long-term tolerability profiles. In the setting of MASLD/MASH, the metabolic safety of PPAR agonists is characterised by a paradoxical interplay between systemic weight changes and hepatic lipid resolution [156].

The safety profile of PPAR agonists in MASLD and MASH is fundamentally determined by the receptor specificity of each agent, the degree of agonistic activity across individual receptor subtypes, and the complex cardiometabolic milieu—comprising insulin resistance, dyslipidaemia, T2DM, and overweight/obesity—that typifies the MASLD population, thereby rendering multidimensional risk assessment indispensable in clinical practice [157].

Among PPARγ agonists, pioglitazone remains the agent supported by the most robust clinical evidence. Narrative reviews and international guidelines consistently recognise its efficacy in improving hepatic steatosis, promoting MASH resolution, and attenuating fibrosis in patients with T2DM; however, they also highlight a tolerability profile characterised by weight gain, fluid retention, peripheral oedema, and increased risk of heart failure. These adverse effects are attributable to PPARγ-mediated activation of epithelial sodium channels (ENaC) within the renal collecting ducts, representing a pharmacological class effect rather than an idiosyncratic toxicity [158,159].

Given that cardiovascular disease represents the leading cause of mortality in MASLD, concerns regarding heart failure risk in patients with impaired cardiac reserve mandate careful baseline cardiac assessment and contraindicate the use of pioglitazone in individuals with New York Heart Association (NYHA) class II heart failure or higher [160].

The landscape of next-generation pan-PPAR agonists is currently dominated by data on lanifibranor, whose safety profile was extensively characterised in the phase 2b randomised NATIVE trial enrolling patients with highly active non-cirrhotic MASH. Treatment discontinuation due to adverse events was remarkably low (3.6% in the 1200 mg arm, 4.8% in the 800 mg arm, and 3.7% in the placebo arm), with the vast majority of treatment-emergent adverse events (TEAEs) being of mild-to-moderate severity. Rates of serious TEAEs were comparable across groups (<4%), and notably, the only two serious TEAEs considered potentially treatment-related both occurred in the placebo group (one case of mild heart failure and one case of urticaria), effectively excluding evidence of acute drug-specific toxicity [100]. Weight gain—identified as the metabolically most clinically relevant adverse event—occurred more frequently with lanifibranor than with placebo. Nevertheless, the secondary exploratory analysis of the NATIVE trial published by Cooreman et al. [153] demonstrated that patients treated with lanifibranor experienced a mean weight increase of 2.5 kg, with 49% of patients gaining ≥2.5% of baseline body weight; importantly, unlike placebo-associated weight gain, this increase was accompanied by concomitant improvements in insulin levels, Homeostatic Model Assessment of Insulin Resistance (HOMA-IR), HbA1c, fasting glucose, triglycerides, HDL-C, ApoB, hs-CRP, ferritin, diastolic blood pressure, and hepatic steatosis. Restoration of adiponectin levels in more than 95% (1200 mg arm) and 86% (800 mg arm) of treated patients—compared with only 10% in the placebo group—correlated with improvement across all hepato-metabolic markers, suggesting that lanifibranor-induced weight gain is “metabolically healthy” and fundamentally distinct from placebo-associated weight gain, which instead correlated with worsening cardiometabolic parameters. This concept of “qualitative weight gain” is of critical clinical relevance in MASLD, where visceral adiposity and insulin resistance—rather than isolated numerical increases in body weight—constitute the principal pathogenic determinants. Crucially, euglycaemic hyperinsulinaemic clamp studies have confirmed that the efficacy of lanifibranor in improving hepatic and peripheral insulin resistance is maintained irrespective of the magnitude of weight change, suggesting that pan-PPAR activation effectively uncouples adipogenesis from metabolic dysfunction [152,153]. In a subsequent phase 2 proof-of-concept study involving 38 patients with T2DM and MASLD randomised to lanifibranor 800 mg or placebo for 24 weeks [152], lanifibranor significantly reduced intrahepatic triglyceride (IHTG) content by 44% compared with 12% in the placebo group while simultaneously improving hepatic, muscular, and adipose tissue insulin sensitivity. The overall safety profile was favourable (“safe, associated with modest weight gain and mild adverse events”), thereby confirming the reproducibility of the NATIVE safety findings in a T2DM/MASLD population. These findings position lanifibranor as an insulin-sensitising agent rather than merely a lipid metabolic modulator, with potentially important implications for cardiovascular risk stratification in MASH, a disease in which cardiovascular events remain the leading cause of mortality [153,161].

Regarding saroglitazar, safety data derive from a combination of controlled trials, real-world studies, and meta-analyses of randomised controlled trials. The meta-analysis by Durga et al. [162], which included seven randomized controlled trials (RCTs) involving patients with cardiometabolic diseases, demonstrated no statistically significant increase in adverse events with saroglitazar compared with comparator therapies (non-significant RR), while documenting significant improvements in triglycerides, total cholesterol, LDL cholesterol, non-HDL cholesterol, HDL cholesterol, and hepatic biomarkers (ALP, GGT). In the prospective randomised controlled phase 4 interventional trial [163], saroglitazar 4 mg was generally well tolerated in a real-world population characterised by extensive metabolic comorbidity, while significantly improving all assessed parameters of steatosis (CAP), liver stiffness (LSM), liver enzymes, glycaemic indices, and lipid profiles; overall safety was described by the investigators as “generally good” [163]. These large-scale findings are consistent with previous real-world evidence. Chaudhuri et al. [164] in a prospective cohort of 76 patients with MASLD—including 11 with compensated cirrhosis—treated with saroglitazar 4 mg for 52 weeks, reported significant improvements in LSM, CAP, ALT, AST, HbA1c, LDL cholesterol, total cholesterol, and triglycerides, without changes in body weight or serious adverse reactions, while maintaining a favourable safety profile even in the cirrhotic subgroup under close monitoring. Similarly, Rajesh et al., in a prospective pilot interventional study involving 85 patients with MASLD and diabetic dyslipidaemia treated for 12 weeks, observed no treatment-related adverse events [165]; Gupta et al. [166], in a prospective multicentre real-world analysis of 50 patients with MASLD over six months, further confirmed the favourable hepatic and metabolic safety profile of the compound. The absence of fluid retention, peripheral oedema, weight gain, and heart failure risk in the safety profile of saroglitazar—thereby clearly distinguishing it from pioglitazone—is mechanistically coherent with the predominance of PPARα over PPARγ activity. Moderate PPARγ activation appears insufficient to induce the adverse class effects characteristic of pure thiazolidinediones [162,167].

From a cardiovascular perspective, the MASLD setting necessitates particular caution: advanced forms of MASLD/MASH are associated with markedly elevated risk of atherosclerotic cardiovascular events, and agents capable of improving the global cardiometabolic profile—even in the absence of formal cardiovascular outcome data—may plausibly reduce residual cardiovascular risk [161].

The network meta-analysis by Zhong et al. confirmed that the majority of pharmacological agents investigated for MASLD—including PPAR agonists—demonstrate acceptable safety profiles at 24 weeks, with efruxifermin exhibiting the least favourable safety profile among the tested compounds (OR for adverse events 0.32, 95% CI 0.06–0.70 vs. placebo), but without any specific safety signal attributable to PPAR agonists [168].

The narrative review by Koullias et al. [167] further emphasised that, within the current therapeutic landscape of MASLD, pan-PPAR agonists such as lanifibranor—through the combined activation of PPARα (fatty acid β-oxidation), PPARδ (reduction in hepatic inflammation and mitochondrial protection), and PPARγ (insulin sensitisation and adipose tissue redistribution)—simultaneously target the principal pathogenic drivers of MASH without the limiting class effects associated with selective agonists. Similarly, Yoneda et al. underscored that the mechanistic rationale for pan-PPAR agonism in MASLD/MASH resides in its synergistic effects on steatosis, inflammation, and fibrosis, supported by the histological superiority observed in the NATIVE trial, while the currently available safety data do not suggest emerging toxicity signals associated with the pan-PPAR profile compared with selective agonists [169].

Conclusively, the available evidence indicates that lanifibranor, as a pan-PPAR agonist, currently exhibits the most sophisticated and potentially advantageous metabolic safety profile in MASH, characterised by metabolically healthy weight gain and global cardiometabolic improvement, in the absence of treatment-related serious TEAEs in phase 2b trials, although confirmation from phase 3 and long-term studies remains awaited.

### 6.2. Overviewing Safety Profiles of PPAR Agonists in Immune-Mediated Cholestatic Liver Diseases

In cholestatic populations, the metabolic safety of PPAR agonists is predominantly reflected in favourable lipid modulation. The safety profile of PPAR agonists in PBC has been characterised with increasing methodological rigour across both quantitative syntheses of randomised controlled trials and dedicated phase 2 and 3 clinical programmes. A meta-analysis of 17 RCTs conducted by Saeedian et al. [170], encompassing a total of 1219 patients with PBC, demonstrated that the combination of PPAR agonists and UDCA was associated with significant reductions in ALT, although overall hepatic safety remained comparable to UDCA monotherapy. Importantly, estimation of the risk of serious adverse events (SAEs) was limited by substantial methodological imprecision, reflected by wide confidence intervals that constrained the robustness of the absolute risk estimate. Similarly, the meta-analysis by Liao et al. [171], focused on fibrosing cholangiopathies, confirmed this overall safety pattern, revealing no statistically significant differences in rates of serious adverse events between patients treated with PPAR agonists and those receiving placebo, thereby supporting the overall class tolerability of these agents. Nevertheless, the distinction between molecule-specific profiles remains essential.

Elafibranor, evaluated by Schattenberg et al. [139] in a phase 2 randomised trial involving 45 patients with incomplete UDCA response, demonstrated a favourable safety profile during 12 weeks of treatment, with non-serious adverse events of mild-to-moderate intensity, including headache, fatigue, and gastrointestinal symptoms. Crucially, however, two patients receiving elafibranor developed elevations in transaminases requiring treatment discontinuation, thereby suggesting a potential hepatotoxicity signal not reproduced with pure PPARα agonists (fibrates), which conversely tend to reduce ALT through modulation of hepatic lipid metabolism. Unlike PPARγ agonists (e.g., pioglitazone), both elafibranor and seladelpar are devoid of cardiometabolic adverse effects—namely fluid retention, peripheral oedema, and heart failure—related to γ-receptor activation, thereby sharply distinguishing these agents from thiazolidinediones in the context of PBC.

Seladelpar, evaluated by Hirschfield et al. in the phase 3 ENHANCE trial, demonstrated significant reductions in ALT compared with placebo (5 mg arm: −23.4%, *p* = 0.0008; 10 mg arm: −16.7%, *p* = 0.03), without any treatment-related serious adverse events, thereby supporting a reassuring hepatic safety profile [130]. From an extrahepatic perspective, both next-generation agents have shown favourable effects on pruritus. Elafibranor neither induced nor exacerbated pruritus in treated patients, while individuals with baseline pruritus reported symptomatic improvement at the end of treatment [139].

Seladelpar, by contrast, demonstrated significant reductions in pruritus numerical rating scale (NRS) scores in the ENHANCE trial compared with placebo (10 mg arm: −3.14 vs. −1.55 with placebo, *p* = 0.02) [130], reflecting an anticholestatic antipruritic effect that represents a clinically meaningful advantage over obeticholic acid, which is known to exacerbate pruritus. Regarding renal function, long-term safety, and treatment discontinuation rates, currently available evidence remains limited by methodological heterogeneity and generally short follow-up durations across systematic reviews.

The meta-analysis by Saeedian et al. identified substantial imprecision in SAE estimation, with funnel plots suggestive of publication bias for bilirubin outcomes and also documented marked heterogeneity in ALT and AST changes, thereby limiting confidence in these estimates. Overall, the currently available evidence supports a generally favourable hepatic and extrahepatic tolerability profile for PPAR agonists in PBC; however, the absence of treatment-related serious adverse events within clinical trial cohorts does not exclude the possibility of emerging long-term safety signals. Molecule-specific distinctions, particularly the potential for dose-related transaminase elevations with elafibranor and the differential antipruritic profile of seladelpar, remain essential for individualised risk stratification [170].

### 6.3. Limitations of Current Evidence

Despite the advances documented across the clinical trials discussed above, the current evidence base supporting PPAR agonists in chronic liver diseases—both in PBC and MASLD/MASH—remains affected by substantial methodological, structural, and translational limitations that warrant caution in the interpretation of available findings and in their extrapolation to routine clinical practice.

One of the most critical limitations concerns the profound heterogeneity of clinical trial frameworks across the two disease settings, which renders direct comparisons between compounds and across indications inherently problematic.

In PBC, available trials—including those evaluating elafibranor [139], seladelpar [130,172] and saroglitazar [173]—have adopted composite biochemical endpoints (ALP, bilirubin, composite response), with heterogeneous response thresholds, variable follow-up durations (ranging from 12 weeks to 24 months), and, in some instances, premature study termination—as occurred in the seladelpar extension study for reasons unrelated to the drug’s intrinsic safety profile [172]—thereby compromising dataset completeness.

The meta-analysis by Saeedian et al. [170] explicitly documented substantial statistical heterogeneity for ALT and AST changes, as well as potential publication bias for the bilirubin endpoint, underscoring that pooled estimates should be interpreted cautiously within a GRADE framework.

In MASLD/MASH, the issue of heterogeneity is further amplified by the intrinsic complexity of the disease itself. Available trials involving lanifibranor [100], saroglitazar [134], and elafibranor—which has now exited the MASH therapeutic landscape following the failure of the RESOLVE-IT trial [174]—differ considerably with respect to histological inclusion criteria, scoring systems employed (NAS vs. SAF), definitions of treatment response, proportions of patients with diabetes, and fibrosis stage distribution.

Consequently, all indirect comparisons remain methodologically fragile, and any network meta-analysis [168] is subject to substantial uncertainty related to violations of transitivity assumptions. A second major limitation is the near-complete absence of data on hard clinical endpoints—including progression to cirrhosis, hepatic decompensation, liver transplantation, and liver-related or cardiovascular mortality—across both disease settings.

All registered trials evaluating the PPAR agonists discussed in this review have relied on biochemical (ALP, bilirubin, ALT) or histological (MASH resolution, fibrosis stage improvement) surrogate endpoints as primary outcomes; none have been designed or adequately powered to demonstrate benefit on long-term clinical outcomes.

In PBC, FDA approval of elafibranor and seladelpar was granted through accelerated pathways based on ALP reduction as a surrogate endpoint, with mandatory post-marketing confirmatory studies required [146,175]. Although this regulatory strategy is pragmatically justified by the urgent unmet clinical need, it introduces a fundamental uncertainty regarding the extent to which biochemical improvement translates into reductions in mortality or transplantation risk. In MASH, the ongoing NATiV3 trial evaluating lanifibranor—with 72 weeks of planned treatment—represents the first structured attempt to generate long-term histological data for a pan-PPAR agonist; however, it likewise was not designed around primary clinical survival endpoints [176,177]. A third major limitation concerns the lack of validated biomarker-based stratification strategies for the prospective identification of treatment responders. In MASLD/MASH, the marked phenotypic heterogeneity of the disease—encompassing variability in insulin resistance severity, lipid profile, inflammatory activity, fibrosis stage, coexistence of T2DM, and predominance of visceral adiposity—has not been systematically leveraged to define subpopulations with a higher probability of response to PPAR agonists relative to alternative therapeutic agents [167,176]. None of the analysed trials pre-specified omics-, genomic-, or metabolomic-based approaches aimed at identifying predictive biomarkers of response.

This gap is particularly relevant for saroglitazar, whose approval for MASH in India is based on populations with potentially distinct metabolic characteristics compared with European and North American cohorts [164,165], thereby limiting the generalisability of currently available evidence. Similarly, in PBC, the absence of validated immunological or inflammatory biomarkers capable of predicting responsiveness to specific PPAR agonists—as opposed, for example, to FXR agonists or bile acid analogues—precludes biologically driven, patient-specific treatment selection and confines therapeutic stratification largely to clinical criteria alone [178,179].

Pending the availability of phase 3 trials incorporating robust clinical endpoints, long-term extension programmes, and translational studies specifically dedicated to phenotypic stratification, the current evidence supporting PPAR agonists in CLD should be regarded as highly promising yet structurally incomplete, with a still substantial gap between demonstrated biochemical or histological benefit and definitive proof of modification of the natural history of disease.

## 7. Future Perspectives in Pharmacological Modulation of PPARs

### 7.1. Precision Hepatology: From One-Size-Fits-All to Tailored Approaches

The emergence of precision medicine in hepatology represents a paradigmatic transition that fundamentally redefines the rationale for the use of PPAR agonists in CLDs, shifting the conceptual focus from class-based biochemical efficacy alone toward patient phenotypic stratification, multi-system omics integration, and molecular modulation of therapeutic pathways according to the individual biological profile [180].

This transition has been enabled by the conceptual framework of systems biology, which conceptualises liver disease as a state of organ dysfunction emerging from interconnected molecular networks involving metabolism, immunity, the microbiota, and fibrosis [181], thereby necessitating the integration of multi-tissue, multi-omics, and environmental data as a prerequisite for effective therapeutic stratification [182,183,184].

Within this framework, the gut–liver axis has emerged as one of the most relevant pathophysiological mediators in MASLD/MASH. Alterations in gut microbiome composition—including depletion of butyrate-producing taxa and expansion of dysbiotic microbial populations—modulate hepatic inflammation through the portal circulation and amplify hepatocellular lipotoxicity [185,186,187,188,189], with direct implications for stratifying patients who may benefit from PPAR agonists, since therapeutic responsiveness is likely influenced by microbiome composition and intestinal permeability. Integrated multi-omics approaches have begun to define MASLD-specific molecular signatures, such as the palmitoylation system and regulation of COX6A1 as key metabolic nodes in mitochondrial dysfunction [185], as well as multilayered signatures linking environmental exposome, lipid metabolism, and steatotic phenotype [188,189,190].

Beyond the compositional changes of the gut microbiota, increasing attention has focused on microbiota-derived metabolites as functional mediators of gut–liver communication [186]. Among these, tryptophan-derived indole metabolites act as endogenous ligands of the aryl hydrocarbon receptor (AhR), a ligand-activated transcription factor involved in xenobiotic sensing, intestinal barrier integrity, and immune homeostasis [187]. Emerging evidence suggests a functional crosstalk between AhR and PPAR signaling pathways, with convergence on the regulation of lipid metabolism, macrophage activation, oxidative stress, and inflammatory cytokine production [188]. This integrated signaling network may represent an important mechanistic link through which environmental exposures, diet, and microbial metabolism influence hepatic immunometabolic responses.

However, although these findings provide an attractive biological framework, current evidence remains largely preclinical, and AhR should presently be regarded as an upstream mechanistic integrator rather than a therapeutic target with a level of clinical maturity comparable to FXR- or PPAR-directed therapies.

In PBC, the advent of second-generation PPAR agonists—elafibranor and seladelpar, respectively, the first FDA-approved dual PPARα/δ agonist and the first selective PPARδ agonist for this indication [189]—has inaugurated an era of therapeutic stratification based not only on biochemical response to UDCA but also on clinical, biochemical, and molecular features predictive of differential response to second-line therapeutic options. The systematic review and meta-analysis by Dar et al. [190], together with post hoc analyses from the ELATIVE study [191], demonstrated that baseline ALP values significantly modulate the probability of response to elafibranor, with patients exhibiting higher baseline ALP levels achieving superior composite response rates. These findings support the use of pre-treatment biochemical stratification as a therapeutic positioning tool.

Schattenberg et al. [192] further emphasised that the identification of PBC subpopulations characterised by distinct activation profiles of biliary inflammatory, immunological, and fibrotic pathways—potentially accessible through transcriptomic analysis of liver biopsy specimens or circulating cytokine profiling—represents the most urgent frontier for a precision-medicine application of PPAR agonists in PBC.

The dimension of pruritus, as a patient-centred endpoint of substantial translational relevance, is now being quantitatively characterised through the ITCH-E study [193] and through elucidation of the IL-31-mediated mechanisms specifically modulated by seladelpar [194], thereby opening the possibility of immunological pruritus biomarkers as predictors of differential therapeutic response between agents.

Italian Delphi consensus recommendations [195] have formally recognised the need for structured quality measures within the therapeutic pathway of PBC, including systematic assessment of second-line treatment response as a component of routine clinical monitoring. Fiorucci et al. [196], Jamal et al. [197], and Ruiu et al. [198] collectively delineate a framework for positioning second-line therapies in PBC in which the choice between PPAR agonists, obeticholic acid, and future combination regimens should be informed by patient-specific characteristics—including baseline pruritus, fibrosis stage, metabolic profile, and biochemical response—rather than by standardised prescribing algorithms.

In MASLD/MASH, the phenotypic complexity of the disease—which integrates insulin resistance, visceral obesity, atherogenic dyslipidaemia, T2DM, genetic predisposition, and hormonal influences—necessitates a precision-medicine approach grounded in metabolic clustering of patients as a prerequisite for the rational positioning of PPAR agonists [199,200]. The contributions of Ma et al. [201] and Karapanagiotidi et al. [202] demonstrated that genetic variants in PNPLA3, TM6SF2, and HSD17B13 modulate the risk of MASLD progression and likely influence pharmacological responsiveness to PPAR agonists, suggesting that pre-treatment genotyping may eventually inform therapeutic selection. Metabolic heterogeneity in MASLD is further amplified by sex-specific variables, with sexually dimorphic response profiles to PPARγ agonists in men and women related to adipose tissue distribution and oestrogenic signalling [203], as well as by browning phenomena in white adipose tissue modulated by PPARα and PPARγ agonists [204], whose differential therapeutic relevance across distinct MASLD phenotypes remains to be systematically explored.

The pan-PPAR agonism of lanifibranor—which simultaneously integrates fatty acid β-oxidation (PPARα), skeletal muscle oxidative metabolism and anti-inflammatory effects (PPARδ), and adipose tissue insulin sensitisation (PPARγ) [157]—provides a compelling mechanistic rationale for use in MASLD populations characterised by greater metabolic complexity, as reflected by the global cardiometabolic improvements [153].

The incorporation of PPAR agonists into combination therapeutic regimens—including GLP-1 receptor agonists, SGLT2 inhibitors, resmetirom, or anti-fibrotic agents—represents a major priority for future clinical development [204,205,206] to achieve synergistic benefits across steatosis, inflammation, fibrosis, and cardiovascular risk domains that no single agent can comprehensively address in isolation [167,205,206].

Fenofibrate, historically the most extensively studied PPARα agonist, has recently been re-evaluated, documenting hepatic benefits in MASLD [207], thereby supporting the hypothesis that selective PPARα agonists may eventually find a role in specific combination strategies beyond their traditional cardiometabolic indications. Durairajan et al. [208] and Qiu et al. [209] further extend the therapeutic perspective of PPAR agonists to adjacent disease contexts, including alcohol-related liver disease, highlighting that the pleiotropic pharmacodynamic properties of these agents render them potentially relevant across broader CLD phenotypes beyond currently approved indications.

Musculoskeletal complications of advanced MASLD, including osteosarcopenia and skeletal muscle loss, are also emerging as therapeutically relevant concomitant targets that PPARα/δ agonists may modulate through systemic anti-inflammatory and muscle metabolic effects [210]. Ferdous and Ferrell [211], together with Suresh et al. [212], contextualise the role of PPAR agonists in MASLD associated with T2DM as part of an integrated therapeutic strategy that must simultaneously address glycaemic control, hepatic lipotoxicity, and residual cardiovascular risk, reinforcing the concept that identification of the metabolically complex patient represents the central challenge for the precision-medicine implementation of these agents.

Loss or epigenetic dysregulation of PPAR signalling during CLD progression represents one of the most relevant yet therapeutically underexploited pathogenic mechanisms. Theys et al. [213] demonstrated that loss of PPARα function is associated with epigenetic dysregulation of lipid homeostasis, promoting ferroptosis and pyroptosis in MASLD and thereby linking transcriptional silencing of PPARα to lipotoxic cell death pathways that amplify inflammation and fibrotic progression.

Zaiou [214] documented that PPARγ functions both as a target and as a regulator of epigenetic mechanisms in MASLD/MASH, whereby alterations in DNA methylation and histone modifications modify transcriptional receptor sensitivity and influence responsiveness to pharmacological agonists, thereby opening the possibility of personalised epigenomics as a predictive tool for individual drug responsiveness. Gong et al. systematically demonstrated that PPARα regulates hepatic fibrosis through multiple mechanisms—including modulation of HSC activation and suppression of pro-fibrotic pathways—and that loss of PPARα expression in advanced fibrotic hepatocytes reduces responsiveness to PPARα agonists, suggesting a stage-dependent therapeutic responsiveness [215]. Liu et al. further characterised ferroptosis in advanced MASH as a convergent mechanism of lipotoxicity intersecting with PPAR pathways, thereby providing a biological rationale for the use of PPARα agonists before irreversible compromise of the hepatocellular epigenome occurs [216]. Conceptualised nuclear receptors—including PPARs—as molecular hubs integrating metabolic, immune, and oncogenic signalling, emphasising that their epigenetic dysregulation contributes to the transition toward pro-inflammatory and pro-neoplastic phenotypes that PPAR-targeted pharmacology may counteract only if applied within appropriate temporal therapeutic windows.

The palmitoylation system—recently identified by Yu et al. [185] as a regulator of hepatocellular function in MASLD through Cytochrome c Oxidase subunit 6A1 (COX6A1)—represents a potential molecular interlocutor of PPAR signalling that could be exploited in future combinatorial therapeutic strategies.

Baumert et al. [217] demonstrated that environmental exposure to perfluorinated compounds disrupts hepatic metabolic function through mechanisms involving dysregulation of PPAR pathways, thereby highlighting the exposome as a variable capable of modifying individual therapeutic responsiveness and one that precision hepatology will need to systematically integrate into future therapeutic algorithms.

In this scenario, artificial intelligence (AI) and high-resolution digital phenotyping constitute the enabling technological infrastructure required for the practical implementation of precision medicine in evaluating response to PPAR agonists in chronic liver diseases. Zheng et al. documented the multimodal applications of AI in hepatology—from automated diagnosis to radiotemporal analysis of fibrotic progression—as tools capable of overcoming the limitations of traditional serum biomarkers in therapeutic response assessment [218]. Kong et al. [219] further delineated the potential of AI in analysing the intersection between MASLD and cardiovascular risk—two domains simultaneously modifiable by pan-PPAR agonists—thereby opening the possibility of integrated predictive models capable of identifying patients most likely to derive cardiohepatic benefit. Windell et al. developed AI-based algorithms for the detection and characterisation of portal tract features in digital histopathology from MASLD, MASH, and autoimmune hepatitis, demonstrating that digital pathology can stratify lobular and portal inflammatory activity with greater precision than standard human assessment.

These findings carry direct implications for defining histological endpoints in trials evaluating PPAR agonists and for identifying sub-bioptic patterns of therapeutic response [220]. Reinson et al. and Martinou et al. [221] further contextualise the need for validated non-invasive serum fibrosis biomarkers—including ELF score, FIB-4, cytokeratin profiles, and lipidomic signatures—as monitoring tools for therapeutic response to PPAR agonists in future trials that cannot, or should not, rely exclusively on liver biopsy [222].

The integration of radiomic, digital histopathological, transcriptomic, and clinical data into AI models trained on cohorts treated with PPAR agonists—including lanifibranor, saroglitazar, elafibranor, and seladelpar—could ultimately enable the a priori identification of phenotypic characteristics predictive of treatment response and permit dynamic, non-invasive monitoring of therapeutic benefit.

Such an approach would establish a paradigm of personalised pharmacology that the future research agenda in precision hepatology is now called upon to pursue with methodological rigour and translational ambition.

### 7.2. Expanding the Therapeutic Scenarios and Exploring the Role of Combination Therapies

The complex, multifactorial pathophysiology of chronic liver diseases—ranging from the metabolic dysregulation and lipotoxicity of MASLD to the autoimmune-mediated biliary destruction of PBC—increasingly exposes the limitations of monotherapeutic interventions. Current evidence suggests that targeting a single molecular node rarely achieves complete histological resolution or biochemical normalization in the majority of patients. Consequently, the frontier of precision hepatology is shifting toward rational combination regimens and multi-target pharmacological platforms, such as dual and pan-PPAR agonists, to address the “multi-hit” nature of liver injury [223,224].

In the management of MASH, the primary objective of combination therapy is to decouple systemic metabolic stress from intrahepatic inflammatory and fibrotic responses. High-level evidence from recent RCTs and meta-analyses supports the synergy between incretin-based therapies and liver-directed agents [225,226].

Glucagon-like peptide-1 receptor agonists (GLP-1RAs), such as semaglutide, provide potent upstream metabolic unloading by inducing weight loss, improving insulin sensitivity, and reducing the delivery of free fatty acids from adipose tissue to the liver [227]. When paired with PPAR agonists, which directly modulate hepatic lipid β-oxidation (α), anti-inflammatory signaling (δ), and insulin sensitization (γ), the combination addresses both the “first hit” of lipid accumulation and the “second hit” of cellular injury [223,228].

Recent phase 2b data from the ATLAS trial [229] and subsequent analyses [230] demonstrated that while monotherapy with FXR agonists (cilofexor) or acetyl-CoA carboxylase (ACC) inhibitors (firsocostat) yielded modest results, their combination—often integrated with GLP-1 receptor agonists (GLP-1RAs)—resulted in significant improvements in liver steatosis (MRI-PDFF) and non-invasive markers of fibrosis [224,230]. These findings support the concept that targeting complementary pathogenic pathways may provide greater therapeutic benefit than single-agent approaches. The therapeutic landscape of MASH is therefore rapidly evolving beyond PPAR agonism alone, encompassing several mechanism-based strategies, including thyroid hormone receptor-β (THR-β) agonists, GLP-1 receptor agonists, dual GIP/GLP-1 receptor agonists, sodium–glucose cotransporter-2 (SGLT2) inhibitors, ACC inhibitors, FXR agonists, and rational combination regimens. Notably, resmetirom (Rezdiffra), a selective THR-β agonist, became the first pharmacological therapy to receive accelerated FDA approval for adults with non-cirrhotic MASH and stage F2–F3 fibrosis, representing a major milestone in the transition toward disease-modifying therapies [228,231]. In this rapidly evolving therapeutic scenario, PPAR agonists should be viewed as complementary components of a broader mechanism-based treatment paradigm rather than as stand-alone therapeutic strategies.

Their pleiotropic metabolic, anti-inflammatory, and antifibrotic properties make them particularly attractive partners for future combination regimens. Mechanistically, while THR-β agonists primarily enhance hepatocellular lipid catabolism, PPAR agonists additionally modulate inflammatory responses, macrophage activation, and hepatic stellate cell biology, providing a strong biological rationale for combination approaches [192,232].

In the context of PBC, combination strategies aim to stabilize the “bicarbonate umbrella” and reduce bile acid-induced hepatotoxicity. The therapeutic mainstay remains the combination of UDCA with second-line PPAR agonists [233].

Meta-analyses of RCTs confirm that the addition of PPAR-αδ (elafibranor) or selective PPAR-δ (seladelpar) to UDCA significantly increases the rates of composite biochemical response and ALP normalization compared to UDCA monotherapy [190,233].

Emerging paradigms are now exploring “triple therapy” (UDCA + PPAR + FXR agonist) for patients with inadequate responses to dual regimens, leveraging complementary mechanisms of bile acid synthesis inhibition and enhanced biliary export [192]. Rather than competing therapeutic strategies, FXR agonists and PPAR agonists should be regarded as mechanistically complementary approaches targeting distinct but interconnected aspects of cholestatic liver disease, thereby providing a strong rationale for future combination regimens.

Also, in MASLD/MASH, the evolution of “combination-in-a-molecule” platforms—specifically dual and pan-PPAR agonists—represents a significant advancement in therapeutic efficiency. Lanifibranor, a pan-PPAR agonist, demonstrated in the NATIVE phase 2b trial that simultaneous activation of all three isoforms achieved histological MASH resolution and fibrosis improvement, a feat that has historically eluded many single-isoform agonists [100,228]. This approach minimizes the pill burden and reduces the risk of drug–drug interactions associated with multi-drug regimens while achieving global metabolic and hepatic benefits [153].

Similarly, the dual PPAR-αγ agonist saroglitazar has shown robust efficacy in real-world Indian cohorts, improving liver stiffness and steatosis without the profound weight gain typically associated with pure γ-agonists [166,234].

Multi-target strategies are increasingly guided by precision medicine frameworks that integrate multi-omics data and AI-driven radiomics [218,235]. Identification of “at-risk” MASH patients (F2-F3 fibrosis) through non-invasive tests (NITs) like VCTE and MRE allows for the early initiation of these broad-spectrum agents [224,227]. Furthermore, the identification of genetic variants (e.g., *PNPLA3* I148M) and microbial signatures is beginning to inform the sequential versus simultaneous application of therapeutic combinations [235]. For example, patients with high pruritogenic burdens in PBC are increasingly directed toward PPAR-δ agonists (seladelpar) or combinations involving apical sodium-dependent bile acid transporter (ASBT) inhibitors like linerixibat [130,193].

Table 3 summarizes the state of the art of combination therapies and reports the key findings concerning the future generation of PPAR modulators (Table 3).

### 7.3. Future Research Directions: Towards Modern Strategies and New Generation PPAR Modulators

The evolution of PPAR pharmacology in CLD has entered a phase of critical maturation in which future research must address three fundamental and interconnected challenges: the development of next-generation PPAR modulators with improved pharmacological profiles, the validation of dynamic biomarkers capable of guiding individualized therapeutic stratification, and the demonstration of long-term clinical benefit on durable outcome endpoints extending beyond the biochemical or histological surrogates currently adopted in registration trials [127,207]. From the perspective of innovative PPAR modulators, the most relevant evolutionary trajectory is directed toward the pharmacological dissociation between agonistic activity and class-related adverse effects, an objective potentially achievable through three distinct strategies.

The first involves structural chemical modification aimed at stereochemical selectivity: PXL065—deuterium-stabilized (R)-pioglitazone—was evaluated in the phase II DESTINY-1 trial [236], with the aim of preserving PPARγ-mediated insulin sensitization while reducing oxidative metabolism and the systemic adverse effects associated with racemic pioglitazone, achieving histological improvement in patients with MASH alongside a potentially more favorable safety profile. This represents a paradigmatic example of pharmacological optimization of receptor biology rather than abandonment of the therapeutic class itself. The second strategy concerns the development of pan-PPAR agonists derived from unconventional sources: NCPC-626, a microbial metabolite-derived PPARα/β/δ agonist, demonstrated anti-steatotic and anti-inflammatory efficacy in murine models of MASH [237], suggesting that the gut microbiome may not only modulate responsiveness to existing PPAR agonists but also serve as a source of structurally novel PPAR modulators.

The third direction involves combinations of subtype-selective agonists within multi-agent regimens, as demonstrated by Honda et al. [238] in a diet-induced murine model of early MASLD, in which the combination of pemafibrate (PPARα), seladelpar (PPARδ), and pioglitazone (PPARγ) produced synergistic reductions in hepatic lipid accumulation, inflammation, and insulin resistance superior to any individual agent. These findings provide the strongest preclinical proof-of-concept to date for multi-receptor PPAR pharmacology tailored to individual metabolic phenotypes, although clinical translation of this paradigm will require robust evidence of safety and tolerability in well-characterized human cohorts before it can inform clinical practice.

Chiglitazar, a pan-PPAR agonist with predominant α/γ activity, has recently demonstrated, in a randomized phase II study [239], significant reductions in steatosis, triglycerides, and HOMA-IR versus placebo in patients with MASLD, hypertriglyceridemia, and insulin resistance, thereby introducing a novel compound with a differentiated pharmacological profile compared with lanifibranor within the same therapeutic space.

The combination of fibrates with statins—historically constrained by concerns regarding hepatotoxicity and nephrotoxicity, documented by Sobukawa et al. [240]—remains an area of uncertainty requiring dedicated pharmacoepidemiological investigation in MASLD and PBC populations, in whom concomitant indications for these agents are common. In PBC, pharmacological interest is increasingly focused on optimizing the positioning of elafibranor and seladelpar in patients with an incomplete response to UDCA: the systematic review by Dar et al. [190] and the meta-analysis by Tang et al. [233] converge in documenting class efficacy for PPAR agonists while simultaneously highlighting interindividual heterogeneity of response as the principal limitation to broad generalization of outcomes.

Boyer-Diaz et al. [151] demonstrated that lanifibranor improves portal hypertension and hepatic fibrosis in experimental models of advanced chronic liver disease, raising the possibility that pan-PPAR agonists may ultimately find therapeutic application in the management of complications of advanced liver disease beyond their strictly steatotic indications.

The most critical knowledge gaps concern the near-complete absence of data on durable clinical endpoints for all next-generation PPAR agonists.

In MASLD/MASH, no trial involving lanifibranor, saroglitazar [163], or chiglitazar has been designed or powered for endpoints such as transplant-free survival, hepatic decompensation, or cardiovascular mortality—the triad of outcomes that ultimately defines true therapeutic benefit in advanced chronic disease and that GLP-1 receptor agonists and emerging combination therapies [223] are beginning to address. Demonstration of fibrosis regression as a bona fide disease-modifying endpoint remains incomplete for all PPAR agonists, with NATiV3 still awaited to provide 72-week histological data for lanifibranor.

In PBC, open-label extension data with elafibranor extending to three years [241], together with improvements in GLOBE and UK-PBC prognostic scores in the ELATIVE study [242], suggest a favorable impact on disease progression.

However, translation into reductions in mortality and transplantation rates remains to be validated in long-term cohorts. The ColHai registry has demonstrated that predictors of decompensation in cirrhotic PBC patients are clinically identifiable [243], suggesting that response to second-line therapy in this high-risk population should be systematically monitored using validated prognostic scores and integrated into structured follow-up protocols. At present, the two-year open-label extension data for seladelpar [172] represent the longest controlled follow-up available for a next-generation PPAR agonist in cholestatic liver diseases yet remain insufficient for definitive conclusions regarding modification of the natural history of PBC.

Future research agendas must therefore prioritize long-term clinical outcome trials, extension programs incorporating serial biopsy assessment and validated progression measures, combinatorial pharmacology studies integrating histological and molecular endpoints, and the development of multi-omic real-world data platforms capable of enabling dynamic post-marketing surveillance of both safety and effectiveness of PPAR agonists across the full spectrum of metabolic and cholestatic CLD.

## 8. Conclusions

In conclusion, PPAR agonists represent one of the most promising mechanism-based therapeutic strategies in CLDs, owing to their pleiotropic effects on metabolism, inflammation, cholestasis, and fibrogenesis. Current evidence supports their emerging role in MASLD/MASH and cholestatic liver diseases, where selective, dual, and pan-PPAR agonists have demonstrated clinically meaningful benefits across metabolic and hepatic endpoints. Despite the remarkable progress achieved over the past decade, it is important to recognize that most available evidence supporting PPAR agonists is based on surrogate biochemical, histological, or non-invasive endpoints. Improvements in alkaline phosphatase, bilirubin, aminotransferases, steatosis, inflammation, and fibrosis are encouraging and biologically meaningful; however, robust evidence demonstrating reductions in hepatic decompensation, liver transplantation, liver-related mortality, cardiovascular events, or overall mortality remains limited.

Future adequately powered, long-term randomized trials evaluating clinically meaningful outcomes will therefore be essential to define the true disease-modifying potential of PPAR agonists across CLDs. This consideration is particularly relevant for recently approved agents such as elafibranor and seladelpar, whose regulatory approval was based on improvements in validated surrogate biochemical endpoints, while confirmation of long-term clinical benefit awaits ongoing outcome studies. In addition, other substantial challenges remain, including long-term safety, optimal therapeutic positioning, and identification of patients most likely to benefit from treatment. Next therapeutic strategies will likely rely on personalized combination approaches integrating PPAR agonists with agents targeting complementary pathogenic pathways, including THR-β, GLP-1, FXR, and metabolic signaling. Future progress will depend on the integration of multi-omics profiling, digital phenotyping, and artificial intelligence to enable personalized therapeutic algorithms. In this evolving landscape, PPAR-targeted therapies may become a cornerstone of precision hepatology, bridging molecular mechanisms with individualized clinical care.

## Figures and Tables

**Table 1:** Major clinical studies evaluating PPAR agonists in MASLD/MASH.

Study	Drug	Design & Population	Primary Endpoint	Key Results	Ref.
**NATIVE** (NCT03008070)	Lanifibranor *(pan-PPAR α/δ/γ)*	Phase IIb RCT; 247 adults with non-cirrhotic, highly active MASH (800 or 1200 mg/day vs. placebo; 24 weeks).	≥2-point decrease in SAF Activity (SAF-A) score without worsening of fibrosis.	Primary endpoint met: significantly greater SAF-A response with 1200 mg vs. placebo; higher MASH resolution and fibrosis regression, with improved insulin resistance, lipids, and inflammatory markers.	[100]
**NATiV3** (NCT04849728)	Lanifibranor *(pan-PPAR α/δ/γ)*	Phase III RCT; ~1000 adults with non-cirrhotic MASH and fibrosis stage F2–F3; 72 weeks.	MASH resolution and improvement of fibrosis on liver histology.	Enrolment completed (2025); double-blind topline results pending. Earlier blinded/interim signals consistent with improved hepatic and cardiometabolic parameters.	[155]
**GOLDEN-505** (NCT01694849)	Elafibranor *(PPAR α/δ)*	Phase IIb RCT; 274 non-cirrhotic NASH patients (80 or 120 mg/day vs. placebo; 52 weeks); Europe/US.	Resolution of MASH without worsening of fibrosis (modified definition).	Primary endpoint not met overall; NASH resolution favored elafibranor 120 mg in patients with NAS ≥ 4 (post hoc). Improved liver enzymes, lipids, insulin sensitivity, and inflammatory markers; good tolerability.	[135]
**RESOLVE-IT** (NCT02704403)	Elafibranor *(PPAR α/δ)*	Phase III RCT; adults with MASH and fibrosis (120 mg/day vs. placebo).	MASH resolution without worsening of fibrosis (interim/surrogate histological endpoint).	Failed to meet the primary endpoint at interim analysis.	[136,137]
**Saroglitazar US phase 2** (NCT03061721)	Saroglitazar *(PPAR α/γ)*	Phase II RCT; 106 US patients with MASLD/MASH randomized to placebo or saroglitazar 1/2/4 mg; 16 weeks.	Percentage change in serum ALT from baseline at week 16.	Saroglitazar 4 mg significantly improved ALT, liver fat content (MRI-PDFF), HOMA-IR, adiponectin, and triglycerides vs. placebo.	[134]
**Saroglitazar phase 2** (NCT03863574)	Saroglitazar *(PPAR α/γ)*	Phase II RCT in patients with biopsy-defined NASH.	Histological improvement in MASH.	Improvement in histological parameters reported in saroglitazar-treated patients.	[133]

ALT, alanine aminotransferase; HOMA-IR, homeostatic model assessment of insulin resistance; MASH, metabolic dysfunction-associated steatohepatitis; MASLD, metabolic dysfunction-associated steatotic liver disease; MRI-PDFF, magnetic resonance imaging proton density fat fraction; NAS, NAFLD activity score; NCT, National Clinical Trial identifier; PPAR, peroxisome proliferator-activated receptor; RCT, randomized controlled trial; SAF, Steatosis-Activity-Fibrosis score; SAF-A, SAF activity component.

**Table 2:** Major clinical studies evaluating PPAR agonists in primary biliary cholangitis.

Study	Drug	Study Design & Population	Primary Endpoint	Key Results	Ref.
**BEZURSO** (NCT01654731)	Bezafibrate *(pan-PPAR)*	Phase III RCT; 100 PBC patients with inadequate UDCA response; 24 months (add-on to UDCA).	Complete biochemical response.	ALP normalization 67% vs. 2% with placebo; improved pruritus and non-invasive fibrosis markers; creatinine increase observed.	[117]
**Schattenberg et al., 2021** (NCT03124108)	Elafibranor *(PPARα/δ)*	Phase II RCT; 45 PBC patients with inadequate UDCA response; 80 or 120 mg/day vs. placebo; 12 weeks.	Relative change in ALP at week 12.	ALP reduced −48% (80 mg) and −41% (120 mg) vs. +3% placebo; composite response 67–79% vs. 7%; pruritus improved.	[139]
**ELATIVE** (NCT04526665)	Elafibranor *(PPARα/δ)*	Phase III RCT; PBC with inadequate response or intolerance to UDCA; elafibranor 80 mg/day; 52 weeks.	Composite biochemical response (ALP <1.67× ULN, ≥15% ALP decrease, normal bilirubin) at week 52.	Biochemical response 51% vs. 4% placebo; ALP normalization and bilirubin improvement; possible pruritus benefit; AEs: abdominal pain, diarrhea, nausea.	[138]
**Jones et al., 2017** (NCT02609048)	Seladelpar *(PPARδ)*	Phase II RCT; PBC with inadequate UDCA response; seladelpar 50 or 200 mg/day vs. placebo; 12 weeks.	Change in ALP at week 12.	ALP reductions up to ~60% regardless of dose; however, threefold aminotransferase elevations led to early termination.	[127]
**Bowlus et al., 2022** (NCT02955602)	Seladelpar *(PPARδ)*	Phase II, RCT; PBC with inadequate UDCA response; seladelpar 2/5/10 mg/day (titration).	ALP normalization (<1.67× ULN) with normal bilirubin.	Composite response 53% (5 → 10 mg) and 69% (10 mg); well tolerated; no drug-attributed pruritus.	[129]
**ENHANCE** (NCT03602560)	Seladelpar *(PPARδ)*	Phase III RCT; PBC with inadequate UDCA response; seladelpar 5 or 10 mg/day vs. placebo; planned 52 weeks (3-month analysis).	Composite biochemical response at month 3.	Seladelpar 10 mg produced significant anticholestatic effects and reduced pruritus in symptomatic patients.	[130]
**RESPONSE** (NCT04620733)	Seladelpar *(PPARδ)*	Phase III RCT; PBC with inadequate response or intolerance to UDCA; seladelpar 10 mg/day; 12 months.	Composite biochemical response (ALP <1.67× ULN or ≥15% decrease + normal bilirubin) at month 12; pruritus.	Biochemical response 61.7% vs. 20%; pruritus improvement 25% vs. 0%; AEs comparable (86.0% vs. 84.6%).	[125]
**ASSURE** (NCT03301506)	Seladelpar *(PPARδ)*	Open-label, long-term phase III extension; patients rolled over from RESPONSE and legacy seladelpar trials.	Long-term biochemical response, ALP normalization, pruritus, safety.	Response maintained in 70–73% up to 24 months; ALP normalization up to 42% (94% in treatment-naïve at 12 months); durable pruritus improvement; well tolerated.	[131]

AE, adverse event; ALP, alkaline phosphatase; NCT, National Clinical Trial identifier; PBC, primary biliary cholangitis; PPAR, peroxisome proliferator-activated receptor; RCT, randomized controlled trial; UDCA, ursodeoxycholic acid.

**Table 3:** Clinical studies on combination therapies and novel PPAR modulators.

Study	Drug	Design & Population	Primary Endpoint	Key Results	Ref.
**ATLAS** (NCT03449446)	*Cilofexor (FXR agonist) + firsocostat (ACC inhibitor)*—non-PPAR backbone for combination context	Phase IIb RCT; 392 adults with MASH and bridging fibrosis or compensated cirrhosis (F3–F4); multiple mono- and two-drug combinations (cilofexor, firsocostat, selonsertib ± placebo) for 48 weeks.	≥1-stage fibrosis improvement without worsening of NASH at week 48.	Primary endpoint not met; cilofexor + firsocostat combination produced significant NAS reduction, improved steatosis (MRI-PDFF), liver enzymes, and non-invasive fibrosis markers vs. placebo. Pruritus in 20–29% of cilofexor-treated patients.	[229]
**Alkhouri et al., 2022** (NCT03987074)	*Semaglutide (GLP-1RA) + cilofexor + firsocostat*—PPAR-relevant combination	Phase II RCT; adults with MASH and mild-to-moderate fibrosis (F1–F3); semaglutide alone or combined with cilofexor and/or firsocostat for 48 weeks.	Safety; exploratory: change in liver steatosis (MRI-PDFF) and biochemistry.	Combinations generally well tolerated; semaglutide + firsocostat and/or cilofexor produced additional reductions in liver steatosis and biochemistry vs. semaglutide alone. Underpowered for efficacy conclusions.	[230]
**DESTINY-1** (NCT04321343)	PXL065 *(deuterium-stabilized R-pioglitazone; selective non-PPARγ)*	Phase II RCT; 117 adults with MASH (NAS ≥ 4, F1–F3, LFC ≥ 8%); PXL065 7.5/15/22.5 mg/day vs. placebo; 36 weeks.	Relative change in liver fat content (LFC) by MRI-PDFF at week 36.	All PXL065 doses met primary endpoint (LFC −21% to −25% vs. placebo; *p* = 0.008–0.02); 40% at 22.5 mg achieved ≥30% LFC reduction; histological improvement in fibrosis and NAS; favorable safety vs. racemic pioglitazone (reduced weight gain, edema).	[236]

ACC, acetyl-CoA carboxylase; FXR, farnesoid X receptor; GLP-1RA, glucagon-like peptide-1 receptor agonist; LFC, liver fat content; MASH, metabolic dysfunction-associated steatohepatitis; MRI-PDFF, magnetic resonance imaging proton density fat fraction; NAS, NAFLD activity score; NCT, National Clinical Trial identifier; RCT, randomized controlled trial.

## Data Availability

No new data were created or analyzed in this study.

## Abbreviations

The following abbreviations are used in this manuscript:

ABCA1	ATP-binding cassette transporter A1
ABCG1	ATP-binding cassette transporter G1
AF-1	Activation Function-1
AF-2	Activation Function-2
AI	Artificial Intelligence
ALD	Alcohol-Related Liver Disease
ALP	Alkaline Phosphatase
ALT	Alanine Aminotransferase
AMPK	AMP-Activated Protein Kinase
ApoA-I	Apolipoprotein A-I
ApoC-III	Apolipoprotein C-III
AP-1	Activator Protein-1
CBP	CREB-Binding Protein
CLD	Chronic Liver Disease
COX-2	Cyclooxygenase-2
CPT1A	Carnitine Palmitoyltransferase 1A
CRP	C-Reactive Protein
DAMP	Damage-Associated Molecular Pattern
DBD	DNA-Binding Domain
ECM	Extracellular Matrix
ELF	Enhanced Liver Fibrosis
ER	Endoplasmic Reticulum
FDA	Food and Drug Administration
FIB-4	Fibrosis-4 Index
FFA	Free Fatty Acid
GGT	Gamma-Glutamyl Transferase
GLP-1	Glucagon-Like Peptide-1
HCC	Hepatocellular Carcinoma
HBV	Hepatitis B Virus
HCV	Hepatitis C Virus
HDL	High-Density Lipoprotein
HFD	High-Fat Diet
HSC	Hepatic Stellate Cell
IFN-γ	Interferon Gamma
IL	Interleukin
LBD	Ligand-Binding Domain
LDL	Low-Density Lipoprotein
LOXL	Lysyl Oxidase-Like Protein
MASLD	Metabolic Dysfunction-Associated Steatotic Liver Disease
MASH	Metabolic Dysfunction-Associated Steatohepatitis
MCP-1	Monocyte Chemoattractant Protein-1
miRNA	MicroRNA
NF-κB	Nuclear Factor Kappa B
OCA	Obeticholic Acid
PBC	Primary Biliary Cholangitis
PDGF	Platelet-Derived Growth Factor
PIIINP	N-terminal Propeptide of Type III Procollagen
PPAR	Peroxisome Proliferator-Activated Receptor
PSC	Primary Sclerosing Cholangitis
PPRE	Peroxisome Proliferator Response Element
ROS	Reactive Oxygen Species
RXR	Retinoid X Receptor
SAF	Steatosis Activity Fibrosis
sdLDL	Small Dense Low-Density Lipoprotein
SGLT2	Sodium–Glucose Cotransporter-2
SMAD	Small Mothers Against Decapentaplegic
T2DM	Type 2 Diabetes Mellitus
TGF-β	Transforming Growth Factor Beta
THR-β	Thyroid Hormone Receptor Beta
TLR	Toll-Like Receptor
TNF-α	Tumor Necrosis Factor Alpha
UDCA	Ursodeoxycholic Acid
VCAM-1	Vascular Cell Adhesion Molecule-1
VLDL	Very Low-Density Lipoprotein
